# Universal protection against influenza viruses by multi-subtype neuraminidase and M2 ectodomain virus-like particle

**DOI:** 10.1371/journal.ppat.1010755

**Published:** 2022-08-25

**Authors:** Ki-Hye Kim, Zhuo Li, Noopur Bhatnagar, Jeeva Subbiah, Bo Ryoung Park, Chong Hyun Shin, Peter Pushko, Bao-Zhong Wang, Sang-Moo Kang

**Affiliations:** 1 Center for Inflammation, Immunity & Infection, Institute for Biomedical Sciences, Georgia State University, Atlanta, Georgia, United States of America; 2 Medigen, Inc., Frederick, Maryland, United States of America; Chang Gung University, TAIWAN

## Abstract

Annual influenza vaccination is recommended to update the variable hemagglutinin antigens. Here, we first designed a virus-like particle (VLP) displaying consensus multi-neuraminidase (NA) subtypes (cN1, cN2, B cNA) and M2 ectodomain (M2e) tandem repeat (m-cNA-M2e VLP). Vaccination of mice with m-cNA-M2e VLP induced broad NA inhibition (NAI), and M2e antibodies as well as interferon-gamma secreting T cell responses. Mice vaccinated with m-cNA-M2e VLP were protected against influenza A (H1N1, H5N1, H3N2, H9N2, H7N9) and influenza B (Yamagata and Victoria lineage) viruses containing substantial antigenic variations. Protective immune contributors include cellular and humoral immunity as well as antibody-dependent cellular cytotoxicity. Furthermore, comparable cross protection by m-cNA-M2e VLP vaccination was induced in aged mice. This study supports a novel strategy of developing a universal vaccine against influenza A and B viruses potentially in both young and aged populations by inducing multi-NA subtype and M2e immunity with a single VLP entity.

## Author summary

Current influenza vaccine is based on strain-specific neutralizing immunity to hemagglutinin (HA). The effectiveness of seasonal vaccine is unpredictable and could be below 20% due to continuous changes in HA. Thereby, influenza remains a high risk to human health worldwide. Here, we developed a universal influenza vaccine candidate displaying consensus multi-neuraminidase (NA) subtypes and M2 ectodomain (M2e) tandem repeat. A single vaccine entity inducing immunity to conserved M2e and multi subtype NA proteins was found to be effective in conferring broad cross protection against antigenically diverse influenza A and B viruses in young and aged mice. This study provides impactful insight into developing a universal influenza vaccine in young and aged populations.

## Introduction

The efficacy of current influenza vaccination is based on strain-specific neutralizing immunity against hemagglutinin (HA), a highly variable antigenic target. Because of continuous changes in the receptor binding HA head domain, the effectiveness of seasonal vaccination is unpredictable and could be below 20%, as reported during the 2014–2015 influenza season when H3N2 variants emerged [1,2]. Neuraminidase (NA), an antiviral drug target, is the second major glycoprotein in influenza virus and undergoes slower antigenic changes than HA [3,4]. NA phylogeny is clustered into group 1 NA (N1, N4, N5, N8), group 2 NA (N2, N3, N6, N7, N9), and influenza B NA [3]. NA is known to have multiple functions such as in facilitating the release of newly budded virus particles from the infected cells and in cooperating with HA for an effective entry into host cells [5,6]. Pre-existing NA inhibiting (NAI) antibodies to seasonal or recombinant protein vaccination could be an independent correlate of protection, alleviating disease in humans, suggesting NA as a desirable vaccine target [7,8].

Homologous protection by vaccination with NA either in recombinant proteins or in virus-like particles (VLP) was observed in animal studies [9–11]. Also, to a lesser degree, heterologous protection by cross-reactive NA antibodies within the same subtype viruses was reported after NA vaccination in ferrets [10,12] and in mice [9,11], indicating the benefits of NA immunity in broadening cross protection. NA heterosubtypic protection was not observed [9,10], suggesting the limitation of NA standalone vaccination strategies.

The influenza A virus M2 membrane protein contains highly conserved extracellular ectodomain (M2e). Previous studies tested the concept of utilizing M2e as a universal vaccine target and demonstrated that M2e-based vaccines in different platforms could provide broad cross protection in preclinical studies [13–15]. Antibodies and T cells recognizing M2e epitopes were shown to be protective [16–18]. M2e conjugate vaccines were safe in phase 1 clinical trials [19,20]. Despite broadening cross protection, the efficacy of M2e-based vaccines was suboptimal due to the nature of weak and non-neutralizing immunity.

In this study to overcome the limited breadth of NA immunity, we constructed a single entity of VLP presenting multi-subtype consensus NA proteins (m-cNA) including N1 NA, N2 NA, and influenza B NA, as well as M2e repeat consisted of human, swine, avian influenza A M2e (m-cNA-M2e VLP). For the first time, we demonstrated that a singular unit of m-cNA-M2e VLP induced significant protection against antigenically different subtypes (H1N1, H1N2, H3N3, H5N1, H9N2) of influenza A viruses originated from human and avian hosts, as well as both lineages of influenza B viruses. In this study, we demonstrate that a novel strategy of developing a universal vaccine, presenting multi-cNA and M2e immunogens as a single entity, conferred broader cross protection against antigenically different subtypes of influenza A viruses and two lineage B viruses.

## Results

### Preparation of m-cNA-M2e VLP as a single entity universal vaccine candidate

**Fig 1:**
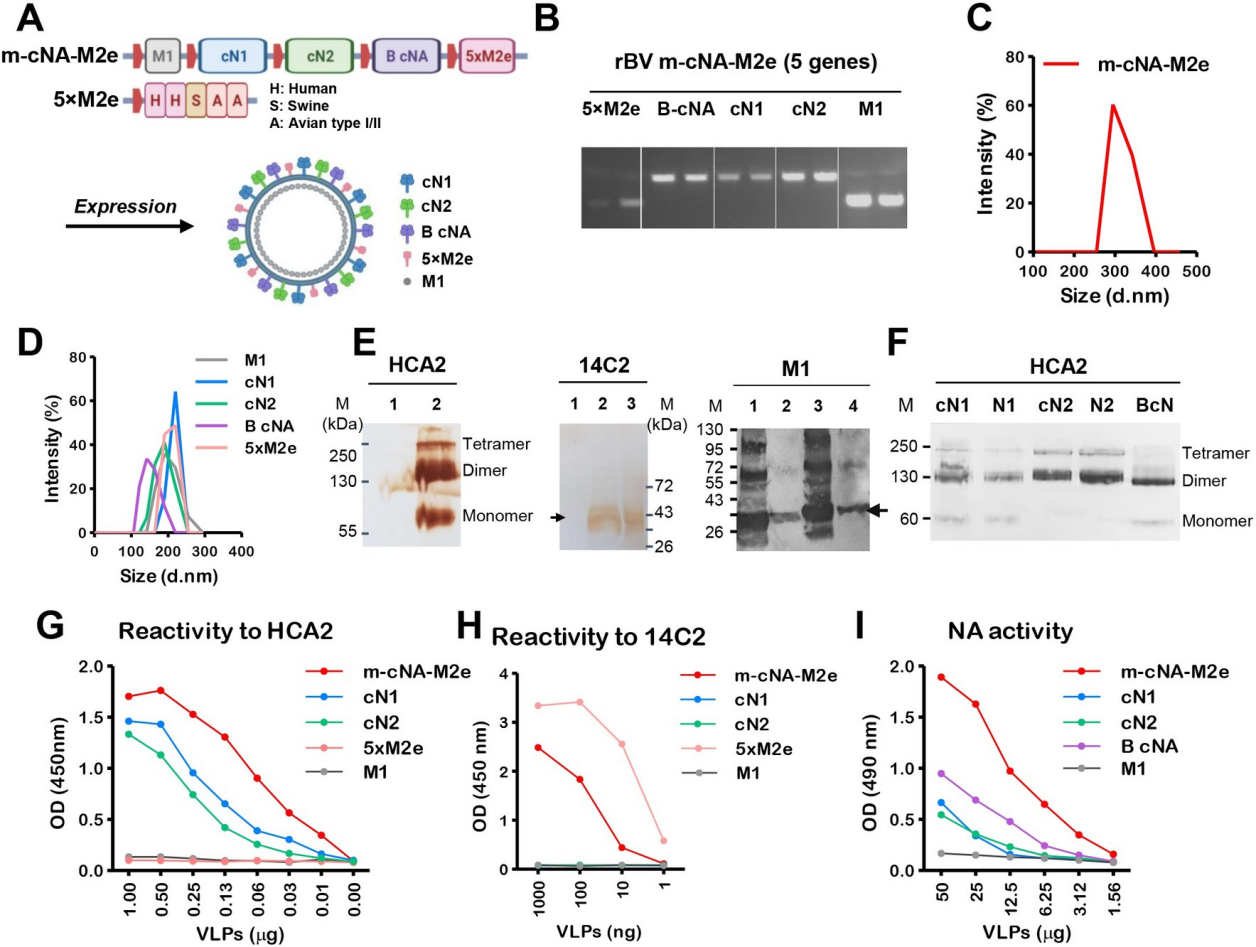
Design and characterization of m-cNA-M2e VLPs containing consensus multi-subtype cNA and tandem repeat 5×M2e. **(A)** Scheme diagram of multi-component m-cNA-M2e VLP vaccine expressing M1, multi subtype cNA (cN1, cN2, and B-cNA), and 5×M2e genes under each polyhedrin promoter (red boxes). (**B)** PCR analysis for confirmation of five genes cloned into the rBV transfer plasmid pFastBac using gene specific primers. (**C and D)** Size distribution of (C) m-cNA-M2e VLP, (D) mono cN1 VLP, cN2 VLP, and 5×M2e VLP. d.nm: diameter in nanometer. (**E)** Western blot analysis of m-cNA-M2e and controls using HCA2 (left) mAb specific for pan NA_222-230_, 14C2 mAb specific for M2e (middle), and M1 (right) specific mAb (ab22396). M: size marker; kDa: kilodalton. VLPs in each line were loaded with 20–30 μg. For HCA2 and 14C2; lane 1 M1 VLP, lane 2 m-cNA-M2e VLP, lane 3 5×M2e VLP. For M1; lane 1 m-cNA-M2e VLP, lane 2 M1 VLP, lane 3 inactivated A/PR8 virus, lane 4 5×M2e VLP. **(F)** Western blot analysis of monomeric VLP samples. VLP and NA protein samples were loaded with 30 μg and 5 μg respectively. cN1: consensus cN1 VLP, N1: NA protein from A/California/04/2009 H1N1 (BEI, NR-19234), cN2: consensus cN2 VLP, N2: NA protein from A/Brisbane/10/2007 H3N2 (BEI, NR-43784), B-cN: influenza B consensus NA VLP. **(G and H)** The reactivity of m-cNA-M2e VLP, mono cNA VLPs and 5×M2e VLP to HCA2 **(G)** or 14C2 **(H)** mAbs by ELISA. **(I)** Functional NA activity of m-cNA-M2e VLP and mono cNA VLPs by ELLA.

Consensus N1 NA (cN1), N2 NA (cN2), and influenza B NA (B cNA) sequences were generated from human isolates (2010–2019) available at NCBI, by using the sequence alignment program (S1 Fig). The highly conserved tandem repeat 5×M2e VLP has been described in prior studies [18]. The rBV vector (pFastBac1) expressing 5 genes (M1-cN1-cN2-B cNA-5×M2e) under each polyhedrin promoter was confirmed for each gene by PCR (Fig 1A and 1B). The particle sizes of multi-component VLPs produced in insect cells were found to be in a range between 250 to 400 nm with an average 325 nm in diameter (Fig 1C), which was slightly bigger than those (averages 142–220 nm) of mono subtype (cN1, cN2, or B cNA) consensus NA VLPs (Fig 1D). The western blot data indicated that NA proteins were presented in heterogeneous multimeric forms on m-cNA-M2e VLP, which includes dimers in majority, monomers, and tetramers, and that 5xM2e and M1 proteins were also packaged into m-cNA-M2e VLP (Fig 1E). Mono subtype cN2 and B cNA VLP preparations showed dimeric forms in majority (Fig 1F).

The m-cNA-M2e construct containing cN1, cN2, and B cNA as well as 5×M2e on the same VLP as a single entity showed reactivity to monoclonal antibody (mAb) HCA-2 specific for pan-NA_222-230_ at higher levels than mono subtype cN1, cN2, and B cNA VLP preparations (Fig 1G). The contents of 5xM2e in m-cNA-M2e VLP were lower compared to 5xM2e monomeric VLP (Fig 1H), probably due to the VLP surface limitation by competitive packaging of multi subtype NA proteins (cN1, cN2, and B cNA) as well as 5xM2e proteins. The monomeric cN1 and cN2 VLP preparations also displayed moderate reactivity to HCA-2 mAb (Fig 1G). In addition, m-cNA-M2e VLP vaccine retained functional activity of NA at higher levels than those observed in mono subtype cN1, cN2, and B cNA VLP preparations (Fig 1I). Taken together, we successfully developed and generated a unique construct of m-cNA-M2e VLP containing consensus multi subtype NA (cN1, cN2, and B cNA) and 5×M2e proteins as a single entity universal vaccine candidate.

### m-cNA-M2e VLP vaccination induces IgG antibodies specific for M2e and NA, and broad NA inhibition activity against influenza A viruses

**Fig 2:**
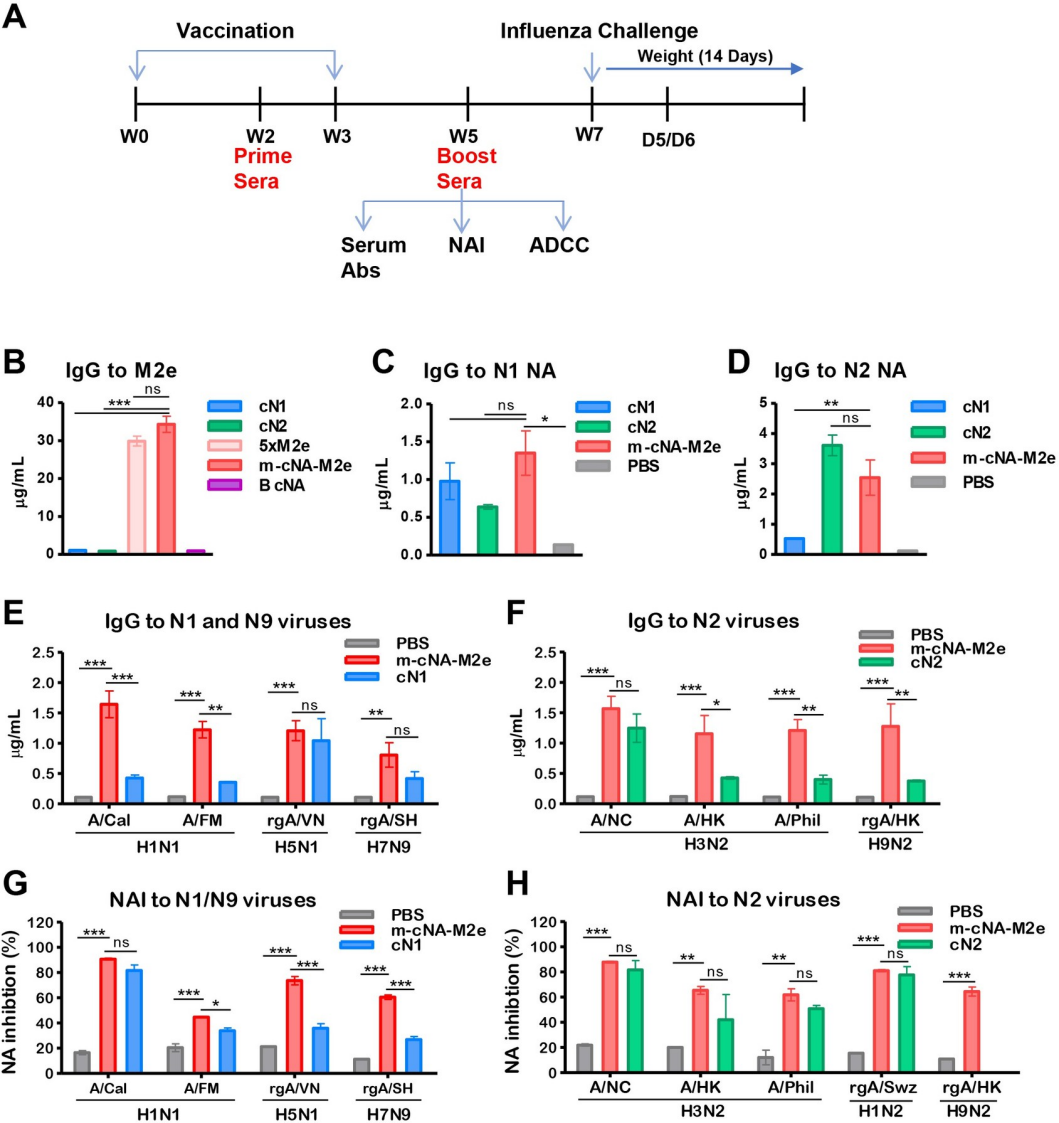
Vaccination with m-cNA-M2e VLP induces IgG antibodies specific for M2e, NA, multi subtype viruses, and broad NA inhibition activity. **(A)** Schematic overview of vaccination of mice, prior to influenza virus challenge. BALB/c mice (n = 10 per group) were intramuscularly immunized twice with each VLP vaccine as indicated. Boost sera (W5) were used for measuring IgG, NAI, and ADCC assay, followed by intranasal (IN) influenza virus challenge at W7 and tissue samples were collected at day 5 or 6 post infection for lung viral titers. cN1: monomeric consensus cN1 NA VLP (3 μg), cN2: monomeric cN2 NA VLP (3 μg), 5xM2e: monomeric 5xM2e VLP (3 μg), m-cNA-M2e VLP: multi-subtype consensus NA (cN1-cN2-B cNA) plus 5xM2e VLP (10 μg). B cNA: monomeric consensus B NA VLP (3 μg). Naïve mice (PBS) were used as a mock control. (**B-F**) IgG antibodies in concentrations as determined by ELISA. IgG antibodies specific for M2e **(B)**, N1 NA (A/Cal/2009 H1N1) **(C)**, N2 NA (A/Brisbane/2007 H3N2) **(D)**, inactivated influenza viruses (**E and F**) in boost immune sera. **(G and H)** Neuraminidase (NA) inhibition activities in 40-fold diluted boost immune sera by ELLA. rgA/PR8-Swz (rgAH1N2): reassortant containing N2 of A/Switzerland/2013 (H3N2) and A/PR8 (H1N1) backbone. All viruses and vaccine groups are as described in Materials and Methods. Data represented as mean ± SEM; statistical significances were performed by (B-D) one-way ANOVA and (E-H) Turkey’s comparison and two-way ANOVA with Bonferroni posttest and indicated as **, *P* < 0.01; ***, *P* < 0.001.

Mice (n = 10 per group) were intramuscularly (IM) prime boost immunized with indicated VLP vaccines at a 3-week interval (Fig 2A). Antisera of m-cNA-M2e VLP boost showed high levels of IgG antibodies for M2e, which are comparable to 5×M2e VLP, but not from the cN1 and cN2 VLP (Fig 2B). The m-cNA-M2e VLP group also induced IgG antibodies specific for N1 and N2 NA proteins at comparable or higher levels, compared to mono cN1 and cN2 VLP (Fig 2C and 2D). Virus binding IgG antibodies were detected at significantly higher levels in antisera of m-cNA-M2e VLP boost than those in mock (PBS) sera, which are specific for H1N1 (A/Cal, A/FM), H5N1 (rgA/VN), and H7N9 (rgA/SH) as well as H3N2 (A/NC, A/HK, A/Phil) and H9N2 (rgA/HK) viruses (Fig 2E and 2F). The cross-reactivity of IgG antibodies was observed to be broader in the m-cNA-M2e VLP group than those in the cN1 or cN2 VLP group (Fig 2E and 2F).

NA inhibition (NAI) titers are likely correlates of protection [7]. Immune sera of m-cNA-M2e VLP exhibited high levels of NAI titers against A/Cal H1N1, rgA/VN H5N1, A/NC H3N2, and rgA/H1N2 (Figs 2G, 2H and S2A). NAI titers against H7N9 (rgA/SH), H3N2 (A/HK, A/Phil), and H9N2 (A/HK) (Figs 2G and S2B) were detected in m-cNA-M2e VLP antisera at a moderate level (Figs 2G, 2H and S2A). In comparison with a mix of monovalent VLPs (VLP mix: cN1 VLP + cN2 VLP + 5xM2e VLP + B-NA VLP, S3 Fig), the m-cNA-M2e VLP group induced similar levels of M2e-specific IgG (S3A Fig) but higher levels of IgG antibodies for N2 NA (S3B Fig), A/California H1N1 virus, and H3N2 viruses (A/Wisconsin, A/Switzerland/2013), and NAI activity against H1N1 and H1N2 virus (S3C–S3G Fig). These results indicate that m-cNA-M2e VLP vaccination induced M2e and NA specific IgG antibodies as well as NA inhibition activity against a broader range of influenza A viruses.

### Single m-cNA-M2e VLP is superior to mono VLPs in inducing broad cross protection against influenza A viruses

**Fig 3:**
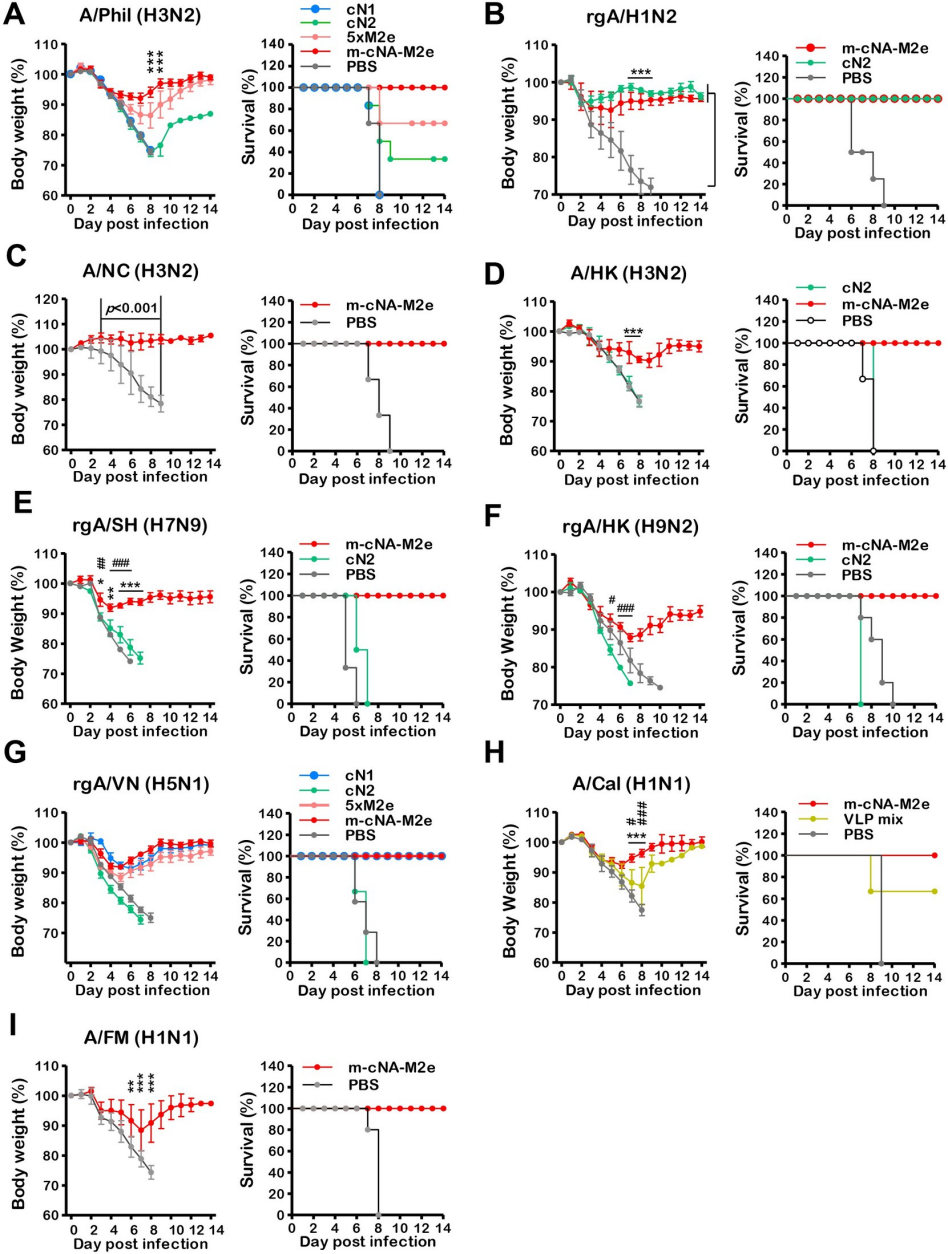
Broad cross protection against different NA subtype influenza A viruses after vaccination. Immunized mice (n = 6 per group) were infected with a lethal dose of different NA subtype influenza A viruses. **(A)** A/Phil/1982 (H3N2) (3xLD_50_, 2.3x10^2^ EID_50_), **(B)** rgA/H1N2 (3xLD_50_, 1.3x10^3^ EID_50_), **(C)** A/NC (H3N2) (A/Nanchang/1995, 2xLD50, 3x10^6^ EID_50_), **(D)** A/HK H3N2 (5xLD_50_, 4x10 EID_50_), **(E)** rgA/SH/2013 (H7N9) (3xLD_50_, 1.1x10^4^ EID_50_), **(F)** rgA/HK/1999 (H9N2) (5xLD_50_, 1.4x10^2^ EID_50_), **(G)** rgA/VN/2004 H5N1 (3xLD_50_, 2.6x10^4^ EID_50_), **(H)** A/Cal/2009 H1N1 (3xLD_50_, 2x10^3^ EID_50_), **(I)** A/FM/1947 H1N1 (3xLD_50_, 8x10^3^ EID_50_). The VLP vaccine dose and groups are the same as in the Fig 2 legend except for the cN2 (cN2 VLP, 10 μg) group in (**B)**. The statistical significances were performed with one-way ANOVA with Tukey’s Multiple Comparison test and indicated as *^,#^ *P* < 0.05; **^,##^, *P* < 0.01; ***^,###^, *P* < 0.001 (compared among the m-cNA-M2e and PBS, monomeric cN, or VLP mix control groups); ns, no significant difference between two compared groups.

We compared the efficacy of m-cNA-M2e VLP, cN1 VLP, cN2 VLP, and 5×M2e VLP after challenge with A/Phil/1982 H3N2 virus at 4 weeks after boost (Fig 3A). The cN1 VLP and mock control mice did not survive. The cN2 VLP group displayed severe weight loss over 20% and survival rates 30%. Substantial weight loss (~15%) and partial protection (60%) were observed in the 5×M2e VLP group (Fig 3A). The m-cNA-M2e VLP group induced most effective protection, with moderate (10%) weight loss (Fig 3A). Both the cN2 VLP and m-cNA-M2e VLP groups were well protected against rgA/H1N2 virus with homologous NA, derived from A/Switzerland/2013 (H3N2) (Fig 3B). The m-cNA-M2e VLP group did not display any weight loss against lethal challenge with A/NC/1995 H3N2 virus whereas the mock control group showed no protection (Fig 3C). Furthermore, m-cNA-M2e VLP despite displaying moderate (10–12%) weight loss was superior to cN2 VLP in conferring effective protection against H3N2 A/HK/1968 (Fig 3D), H7N9 rgA/SH/2013 (Fig 3E), and H9N2 rgA/HK/1999 (Fig 3F) viruses because cN2 VLP was unable to provide protection against the same lethal dose challenge of these viruses (Fig 3D and 3E).

Next, we compared the efficacy of m-cNA-M2e VLP, cN1 VLP, cN2 VLP, and 5×M2e VLP after challenge with rgA/VN H5N1 virus (Fig 3G). The m-cNA-M2e VLP and cN1 VLP groups showed a similar pattern of weight loss (~9%) at days 4 to 6 post challenge whereas 5×M2e VLP exhibited little more (~12%) weight loss (Fig 3G). As expected, the cN2 VLP and unvaccinated groups could not provide any protection against NA heterosubtypic rgA/VN/2004 H5N1 virus.

In addition, we further evaluated whether m-cNA-M2e VLP would have potential advantages over a simple mixture of each mono VLP (VLP mix, Fig 3H). The m-cNA-M2e VLP group showed less weight loss than the VLP mix group (~10% versus 15%) after challenge with A/Cal/2009 H1N1 virus at 4 weeks post boost (Fig 3H). Similarly, the m-cNA-M2e VLP group displayed efficient protection against A/FM H1N1 virus with a moderate level of weight loss (~10%) whereas mock control group did not survive (Fig 3I). Taken together, these data suggest that m-cNA-M2e VLP was superior to monomeric VLP and could be better than VLP mix in conferring a broader window of cross protection against influenza A viruses containing N1, N2, N9.

### Vaccination with m-cNA-M2e VLP induces cross protection against influenza B viruses

**Fig 4:**
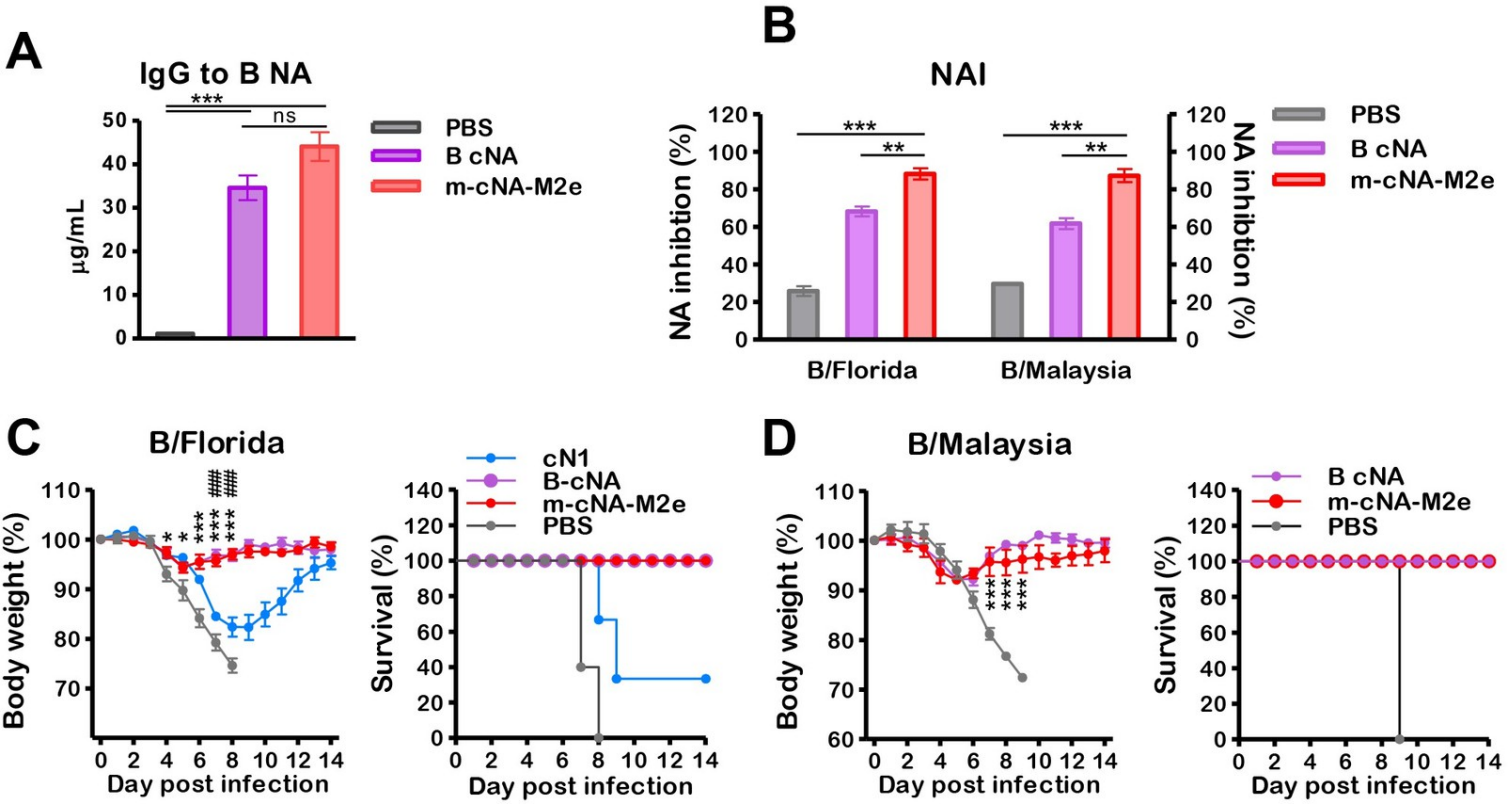
Vaccination with m-cNA-M2e VLP induces cross protection against influenza B viruses. BALB/c mice (n = 10 per group) were intramuscularly prime-boost immunized with m-cNA-M2e VLP (10 μg) or B cNA VLP (3 μg). (A) IgG antibodies specific for flu B NA protein (B/Florida/4/2006) as determined by ELISA. (B) NA inhibition activities against B/Florida/4/2006 and B/Malaysia/2056/2004 viruses in 40-fold diluted boost immune sera by ELLA. (C) Protection against B/Florida/4/2006 (2xLD_50_). (D) Protection against B/Malaysia/2056/2004 (3xLD_50_). The VLP vaccine dose and groups are the same as in the Fig 2 legend. The statistical significances were performed with one-way ANOVA with Tukey’s Multiple Comparison test and indicated as *^,#^ *P* < 0.05; **^,##^, *P* < 0.01; ***^,###^, *P* < 0.001 (compared among the m-cNA-M2e and PBS or monomeric cN control groups).

We investigated whether m-cNA-M2e VLP containing consensus B-NA provides cross lineage protection against influenza B viruses. The IgG antibodies specific for influenza B NA protein were induced at higher levels in boost antisera of m-cNA-M2e VLP group (Fig 4A). NA inhibition activities against Yamagata lineage influenza B/Florida/4/2006 and Victoria lineage B/Malaysia/2056/2004 viruses (Fig 4B) were detected at higher levels in boost immune sera of m-cNA-M2e VLP group compared to those in B cNA VLP and mock control groups.

Furthermore, the mice vaccinated with m-cNA-M2e VLP and B cNA VLP were equally protected against Yamagata lineage B/Florida/4/2006 virus, exhibiting minimum weight loss (~5%) against lethal challenge (Fig 4C). Similarly, the m-cNA-M2e VLP and B cNA VLP groups displayed low weight loss (~5–6%) with 100% survival rates after lethal challenge with Victoria lineage B/Malaysia/2056/2004 virus (Fig 4D). These results support that m-cNA-M2e VLP can provide cross protection against both lineage influenza B viruses in mice, providing further evidence for m-cNA-M2e VLP as a universal vaccine candidate.

### Vaccination of mice with m-cNA-M2e VLP elicits CD4^+^ and CD8^+^ T cell and antibody responses

**Fig 5:**
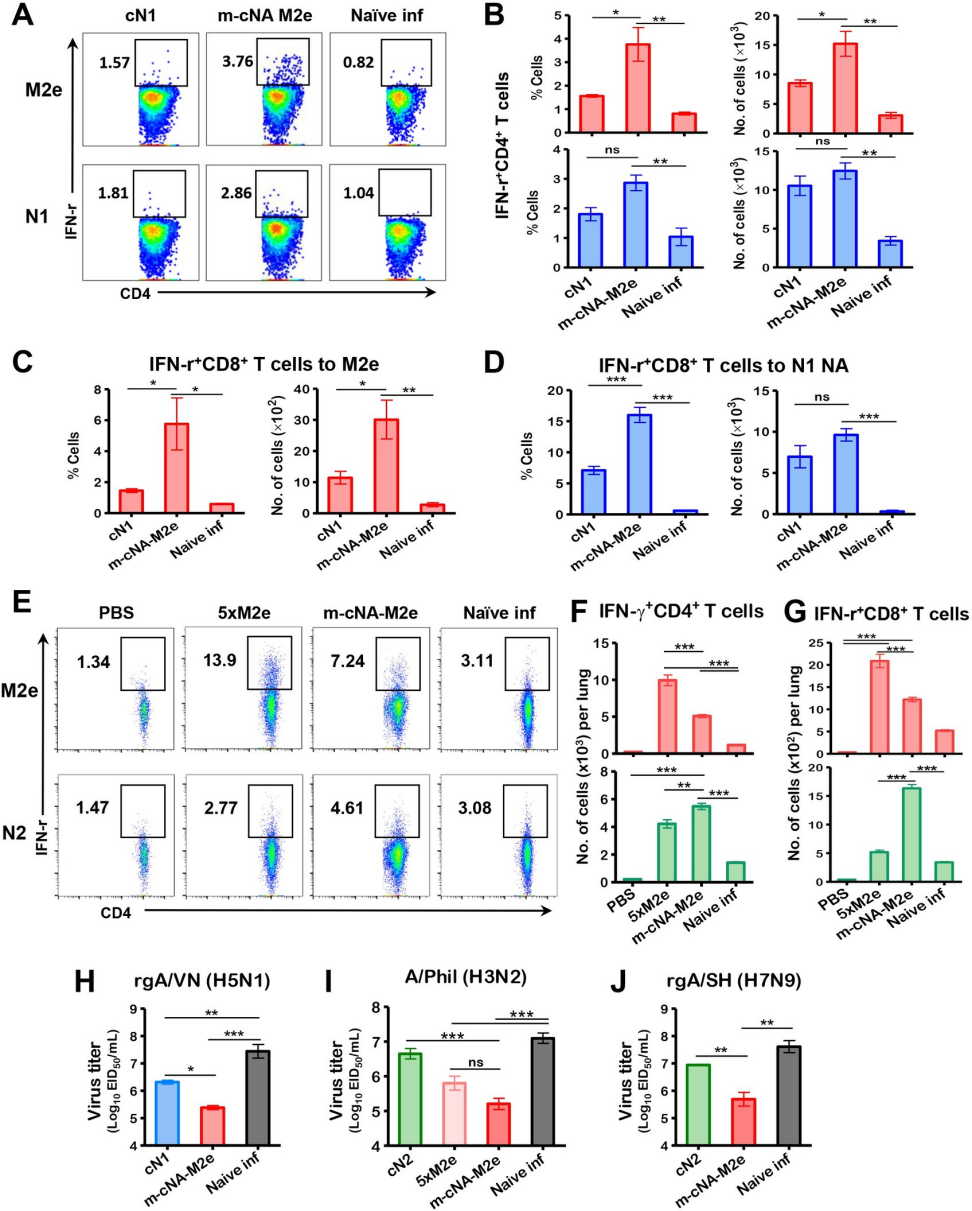
CD4+ and CD8+ T cells responses and diminished lung viral loads by m-cNA-M2e VLP vaccination after virus challenge. Immunized mice (n = 4 per group) were challenged with a lethal dose of (A-D) rgA/VN/2004 H5N1 (3xLD_50_, 2.6x10^4^ EID_50_) and (E-G) A/Phil/1982 (H3N2) virus (3xLD_50_, 2.3x10^2^ EID_50_). (A-G) Effector CD4 or CD8 T cells secreting IFN-γ were analyzed in lung cells harvested on day 6 post infection after *in vitro* stimulation with 5 μg/mL of M2e, N1 NA (A/Vietnam/1203/2004 H5N1) or N2 (A/Brisbane/10/2007) NA pooled peptides. **(A)** Representative flow profiles of IFN-γ^+^CD4 ^+^ T cells gated in percentages. (B) IFN-γ^+^CD4 ^+^ T cells specific for M2e peptide (red bar) or N1 NA peptide pools (blue bar). (C and D) IFN- γ^+^CD8 ^+^ T cells to M2e or N1 NA peptide pools. At day 6 following A/Phil/1982 (H3N2) challenge, (E) representative flow profiles of IFN-γ^+^CD4 ^+^ T cells gated in percentages. (F) IFN- γ^+^CD4 ^+^ T cells specific for M2e (red bar) or N2 NA (A/Brisbane/10/2007) peptide pools (green bar). (G) IFN- γ^+^CD8 ^+^ T cells to M2e (red bar) or N2 NA (green bar) peptide pools. (H-J) Lung viral titers at day 6 post lethal dose infection with (H) rgA/VN/2004 H5N1, (I) A/Phil (H3N2), and (J) rgA/SH (H7N9) by an egg inoculation assay in 10-day embryonated chicken eggs. EID_50_: 50% egg infectious dose. The statistical significances were performed with one-way ANOVA with Tukey’s Multiple Comparison test and indicated as *, *P* < 0.05; **, *P* < 0.01; ***, *P* < 0.001; ns, no significant difference between two compared groups.

Cellular immunity plays an essential role in limiting infection and eventually clearing the virus from the body. Flow cytometry data showed significantly higher levels of IFN-γ^+^CD4 and IFN-γ^+^CD8 T cells upon M2e stimulation of lung cells from m-cNA-M2e VLP vaccinated mice compared to the cN1 VLP group day 6 post rgA/VN H5N1 challenge (Fig 5A–5C). Also, N1 NA stimulated IFN-γ^+^CD8 T cells were induced at higher levels in the m-cNA-M2e VLP group than those after cN1 VLP vaccination (Fig 5D) whereas the levels of NA stimulated IFN-γ^+^CD4 T cells did not show significant difference between the two groups (Fig 5A and 5B). Following A/Phil H3N2 challenge, the m-cNA-M2e VLP group induced significantly high levels of IFN-γ secreting splenocytes and lung cells after *in vitro* stimulation with both M2e and N2 peptides by ELISpot assay, whereas the 5xM2e group showed M2e-stimulated but not NA-stimulated IFN-γ secreting cell spots (S4A and S4B Fig). Consistently, flow cytometry of intracellular cytokine staining showed that M2e and N2-specific CD4^+^ T (Figs 5E and 2F) and CD8^+^ T cells (Fig 5G), which secrete IFN-γ, were substantially increased in the lung of m-cNA-M2e VLP group compared to the naïve infection group. Similarly, the 5xM2e group induced M2e-stimulated IFN-γ^+^ T cells but low or less responsive to NA stimulation as expected (Fig 5E–5G).

We next analyzed *in vitro* IgG producing cell responses induced by immunization with m-cNA-M2e VLP versus mono VLP after A/Phil H3N2 virus challenge. IgG antibodies specific for M2e peptide and N2 NA protein were produced at significantly high levels in culture supernatants of mediastinal lymph node (mLN) and spleen cells (S4C and S4D Fig) from the m-cNA-M2e VLP immunized mice, collected day 6 post challenge with A/Phil (H3N2). In addition, 5xM2e VLP or m-cNA-M2e VLP vaccination induced significantly higher levels of IgG specific for M2e in bronchoalveolar lavage fluids (BALF) and lung lysates than cN2 VLP vaccination and naïve infection (S5A Fig). Meanwhile IgG antibodies specific for N2 NA protein (A/Brisbane H3N2) were detected at significantly higher levels in the m-cNA-M2e VLP and cN2 VLP but not in the 5xM2e VLP (S5B Fig). To assess pulmonary immunopathology, we further measured inflammatory cytokines (IFN-γ and IL-6) in BALF and lung lysates. As expected, the m-cNA-M2e VLP and 5×M2e VLP groups showed significantly lower levels of inflammatory cytokines in BALF and lungs after challenge with A/Phil virus than the cN2 or naive infection group (S5C and S5D Fig).

Consistently, the m-cNA-M2e VLP group showed significantly lower lung viral titers day 6 post challenge with rgA/VN H5N1 (Fig 5H) compared to mock or cN1 VLP, and with A/Phil H3N2 and rgA/SH H7N9 compared to cN2 VLP (Fig 5I and 5J). Taken together, these data suggest that m-cNA-M2e VLP vaccination effectively induced cellular and humoral immunity, contributing to cross protection against different subtypes of influenza A viruses.

### Aged mice vaccinated with m-cNA-M2e VLP effectively induce cross protection against influenza A viruses

**Fig 6:**
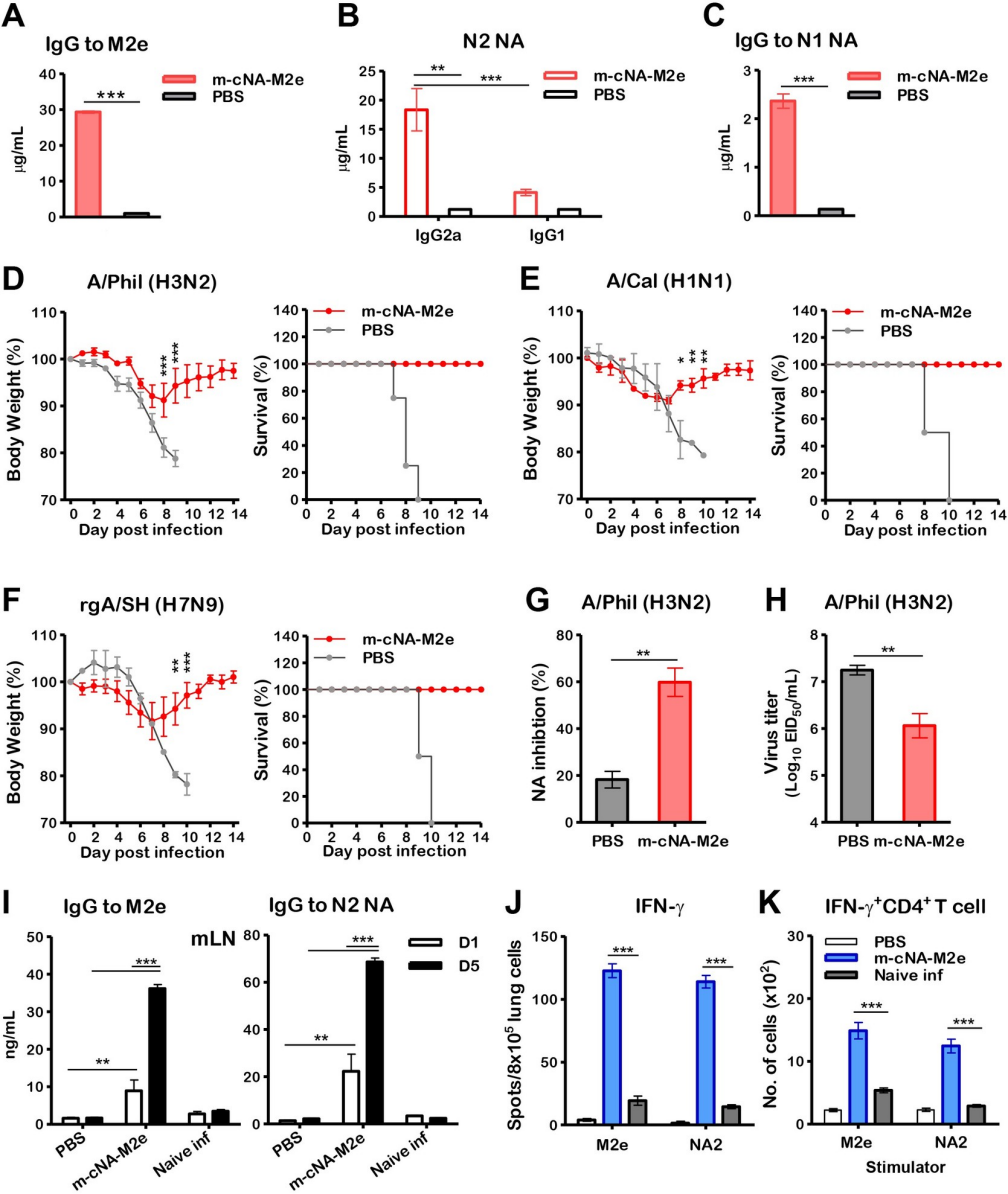
m-cNA-M2e VLP vaccination induces humoral and cellular immune responses and confers cross protection against influenza A viruses in aged mice. BALB/c mice (14-months old, n = 10 per group) were intramuscularly immunized with m-cNA-M2e VLP (10 μg) prime-boost at a 3-week interval. **(A-C)** Concentrations of IgG and isotype antibodies specific for M2e, N2 NA (A/Brisbane/10/2007 H3N2), and N1 NA (A/California/4/2009 H1N1) protein in boost immune sera from aged mice. **(D-F)** Body weight changes monitored daily for 14 days and survival rates after challenge with influenza A viruses. **(D)** A/Phil/1982 H3N2 (3xLD_50_, 7x10 EID_50_), **(E)** A/Cal/2009 H1N1 (3xLD_50_, 2x10^4^ EID_50_), **(F)** rgA/SH/2013 H7N9 (H7 HA, N9 NA from A/Shanghai/2013) (3xLD_50_, 5.6x10^3^ EID_50_). **(G)** NA inhibition activity in 40-fold diluted boost immune sera against A/Phil (H3N2) virus by ELLA. **(H)** Lung viral titers at day 6 post infection with A/Phil (H3N2). **(I)** Antigen-specific IgG levels in mLN cells collected at day 6 post challenge with A/Phil (H3N2) after *in vitro* culture with M2e (4 μg/ml) or N2 NA protein (A/Brisbane/10/2007 H3N2, 200 ng/mL) for 1 (D1) or 5 (D5) days. **(J)** IFN-γ cytokine secreting spots in lung cells after stimulation with 5 μg/mL of M2e or N2 NA (A/Brisbane/10/2007, H3N2) pooled peptide. **(K)** IFN-γ secreting CD4 T cells in lungs after *in vitro* stimulation with 5 μg/mL of M2e or N2 NA pooled peptide by intracellular cytokine staining and flow cytometry. The statistical significances were performed with one-way ANOVA with Tukey’s Multiple comparison test or two-way ANOVA with Bonferroni posttest and indicated as *, *P* < 0.05; **, *P* < 0.01; ***, *P* < 0.001.

Aged mice vaccinated with m-cNA-M2e VLP induced M2e peptide or N2 NA protein-specific IgG in boost immune sera (Fig 6A and 6B), BALF, and lung lysates (S6A and S6B Fig), which were comparable to those in young adult mice (Figs 2A, 2C, S5A and S5B), as well as IgG1, and IgG2a antibodies specific for N2 NA protein (Fig 6B). IgG antibodies specific for N1 NA were induced at lower levels (Fig 6C), which is a similar pattern as in young mice (Fig 2B).

Notably, the aged mice vaccinated with m-cNA-M2e VLP were protected against lethal challenge with A/Phil H3N2 (Fig 6D), A/Cal H1N1 (Fig 6E), and rgA/SH H7N9 (Fig 6F) viruses, showing minimum weight loss (8–10%) with 100% survival rates. In addition, significantly increased NA inhibition activity (60%) (Fig 6G) and reduced lung viral loads (Fig 6H) were observed in the m-cNA-M2e VLP group compared to the mock control.

We further determined humoral and cellular immunity induced by m-cNA-M2e VLP vaccination in aged mice. M2e or NA-specific IgG antibody production in mLN (Fig 6I), IFN-γ secreting cells (Fig 6J), and IFN-γ^+^CD4^+^ T cells (Fig 6K) in lungs were observed at higher levels in aged mice with m-cNA-M2e VLP vaccination compared to mock control at day 6 after A/Phil infection. Consistent with young adult mice (S5C and S5D Fig), m-cNA-M2e VLP-immunized aged mice showed significantly lower levels of inflammatory IFN-γ and IL-6 cytokines after A/Phil challenge (S6C and S6D Fig). Altogether, these data suggest that m-cNA-M2e VLP provides cross protection against different influenza A viruses by inducing humoral and cellular immune responses in aged mice comparable to those in young adult mice.

### Antibody-dependent effector function and cellular immunity induced by m-cNA-M2e VLP vaccination contributes to protection

**Fig 7:**
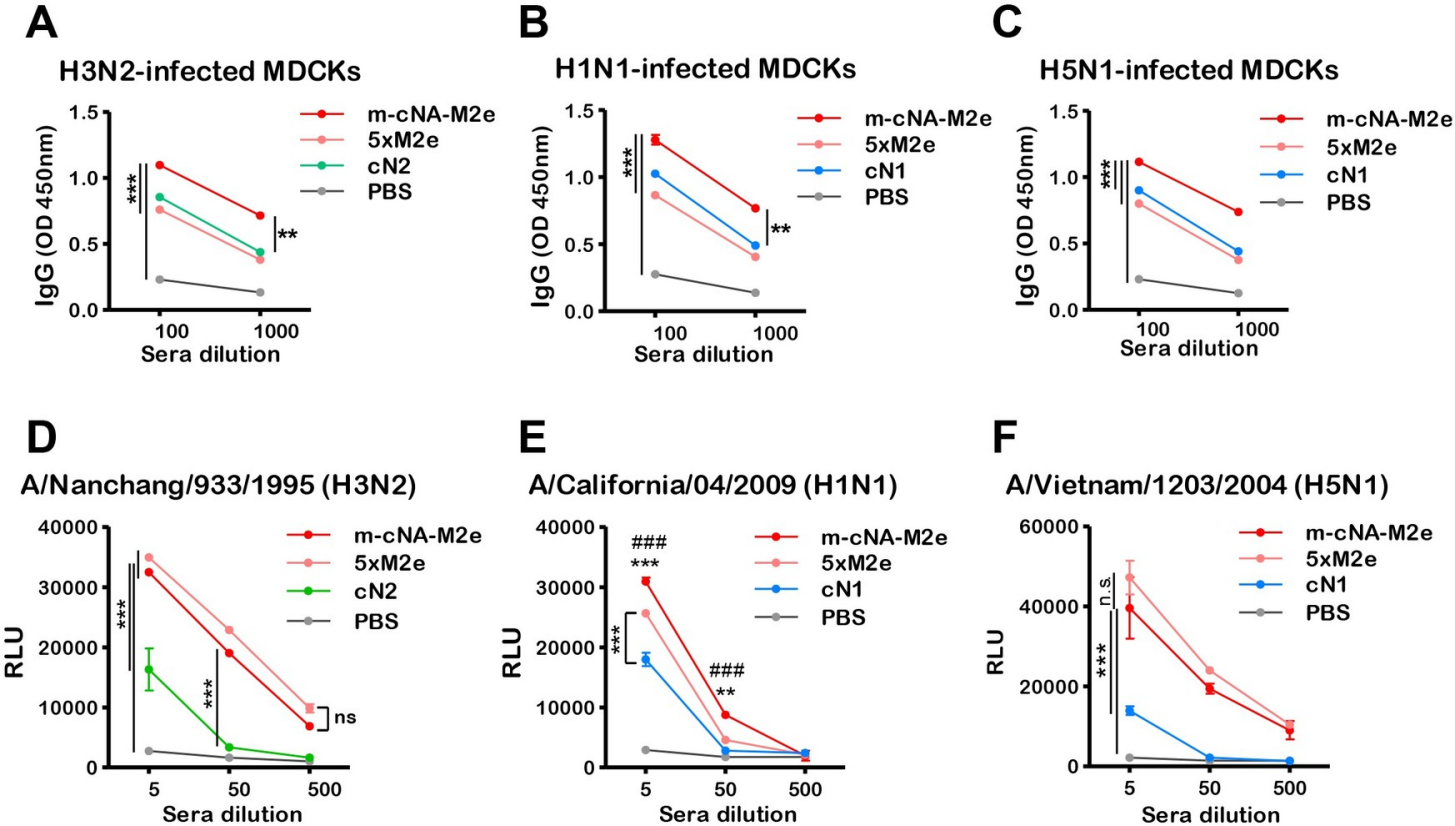
IgG antibodies recognizing cell surface viral antigens and ADCC functional activities by m-cNA-M2e VLP-vaccination. (A-C) IgG antibodies recognizing cell surface viral antigens. MDCKs were infected with influenza A viruses (A/Nanchang/933/1995 H3N2, A/California/04/2009 H1N1, and rgA/Vietnam/1203/2004 H5N1). The levels of IgG binding to virus antigens expressed on MDCKs were determined by ELISA. Binding reactivity of immune sera to H3N2- (A), H1N1- (B), H5N1- (C) infected MDCKs. **(D-F)** ADCC reporter assays of antisera from immunized mice, against MDCK target cells infected with A/Nanchang/933/1995 (H3N2) (D), A/California/04/2009 (H1N1) (E), and rgA/VN/1203/2004 H5N1 (F). Subsequently, the ADCC reporter assay was performed using Jurkat effector cells expressing mouse FcrRIII, and the relative luminescence unit (RLU) was measured. The statistical significances were performed with two-way ANOVA with Bonferroni posttest and indicated as **, *P* < 0.01; ***^,###^, *P* < 0.001 (compared among the m-cNA-M2e and PBS or monomeric cN control groups); ns, no significant difference between two compared groups.

We determined the levels of antibodies binding to the viral antigens on MDCK cells infected with different influenza viruses (Fig 7A–7C). Higher levels of binding IgG were observed in immune sera from the m-cNA-M2e VLP group than those from the mono VLP group (cN1, cN2, and 5×M2e), after infection of MDCK cells with A/NC H3N2, A/Cal H1N1, rgA/VN H5N1 viruses (Fig 7A–7C). In line with these results, ADCC assay in MDCKs which were infected with H3N2 (A/NC), H1N1 (A/Cal), and H5N1 (rgA/VN) viruses, showed stronger induction of the reporter signal of Jurkat cell activation upon treatment with immune sera from m-cNA-M2e or 5×M2e VLP vaccination (Fig 7D–7F). Immune sera of mono VLP (cN1, cN2) vaccination triggered mild induction of the reporter signal, indicating moderate levels of ADCC activity.

**Fig 8:**
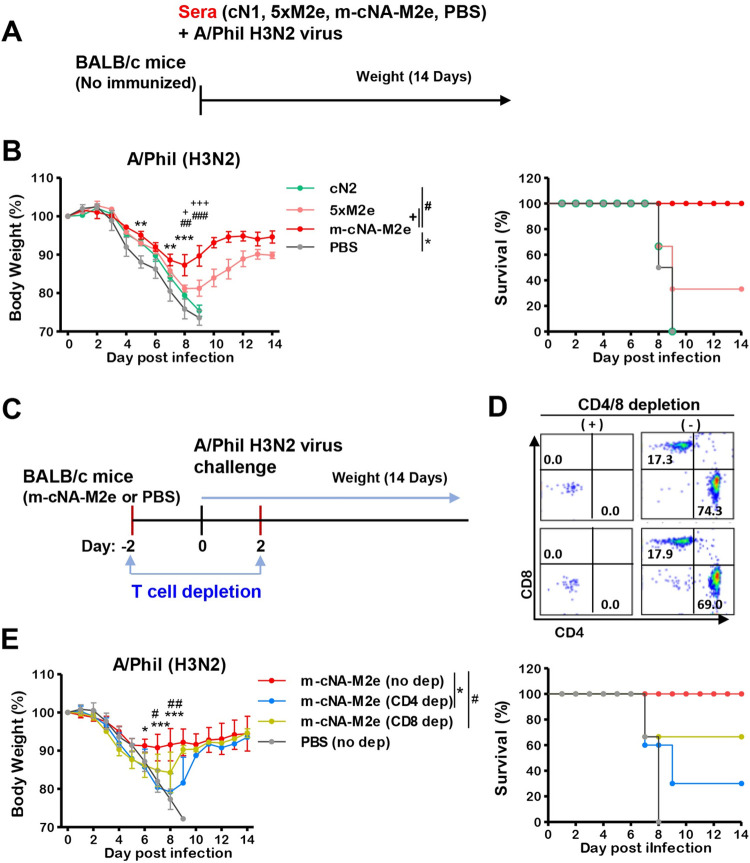
The roles of immune sera and T cell responses in conferring protection. **(A)** Experiment schematic diagram to determine the roles of immune sera. Naïve mice (n = 3 per group) were intranasally infected with a mixture of influenza virus A/Phil H3N2 (4×LD_50_, 3.7x10^2^ EID_50_) and immune sera from the m-cNA-M2e VLP, 5×M2e VLP, cN2 VLP, or naïve control groups. **(B)** Weight changes and survival rates for 14 days. (**C-D**) Contribution of T cells to protection in m-cNA-M2e VLP vaccinated mice. The statistical significances are indicated as *^,#,+^, *P* < 0.05; **^,##^, *P* < 0.01; ***^,###,+++^, *P* < 0.001 and bars mark the comparing groups. (**C**) Experiment schematic diagram to determine the roles of T cells. (**D**) Flow profiles to represent the depletion efficacy of CD4 T cells and CD8 T cells in m-cNA-M2e VLP vaccinated mice (n = 4 per group) by intraperitoneal injection with anti-CD4 (clone GK1.5) or anti-CD8 (clone 53.6.7) antibodies. **(E)** Weight changes and survival rates for 14 days in m-cNA-M2e VLP vaccinated mice with or without T cell depletion after challenge with A/Phil H3N2 virus (4×LD_50_, 3.7x10^2^ EID_50_). The statistical significances are indicated as *^,#,+^, *P* < 0.05; **^,##^, *P* < 0.01; ***^,###,+++^, *P* < 0.001 and bars mark the comparing groups.

To determine the effects of humoral responses in immune sera on cross protection, naïve BALB/c mice were intranasally inoculated with a mixture of A/Phil H3N2 virus and immune sera collected from m-cNA-M2e VLP-, cN2-, or 5×M2e VLP-immunized mice, or naïve sera (PBS). Either cN2 VLP (without M2e specific IgG) or naïve sera (PBS) did not provide protection against A/Phil H3N2 virus as evidenced by severe weight loss (> 25%) and 0% survival rates in naïve mice (Fig 8A and 8B). In contrast, m-cNA-M2e VLP immune sera with M2e specific IgG and NAI titers conferred protection in naïve mice with moderate weight loss (~12%, S6A Fig) and 100% survival rates (Fig 8A and 8B), meanwhile 5×M2e VLP immune sera without NAI titers provided partial protection to naïve mice with more severe weight loss (~19%) and 30% survival rates (Fig 8A and 8B). The overall pattern of protection by antisera was found to be consistent with that of active vaccination, except for the more weight loss in the absence of vaccine-induced T cell immunity.

To investigate whether T cell immunity would contribute to protection of m-cNA-M2e VLP, CD4 T or CD8 T cells were depleted with over 99% efficacy from the mice immunized with m-cNA-M2e VLP prior to A/Phil H3N2 virus challenge (Fig 8C and 8D). Severe weight loss (> 20%, Fig 8E) and low survival rates were observed in the m-cNA-M2e VLP vaccinated mice after CD4 T cell depletion whereas CD8 T cell depletion led to moderate weight loss (~15%) and partial protection compared to the untreated m-cNA-M2e VLP group (Fig 8E). Altogether, these results indicate that CD4 T cells play more protective roles than CD8 T cells in providing protection in the m-cNA-M2e VLP vaccinated mice; furthermore, that humoral immune responses, including ADCC and NAI activity antibodies, contribute to broad cross protection in m-cNA-M2e VLP vaccinated mice.

## Discussion

NA contents in seasonal vaccines are low, variable, and not standardized [9,21]. In addition, due to competition of intravironic HA and NA antigens within the same particle, strain-specific HA immunity is dominant over NA in both T- and B-cell responses to influenza vaccination [22,23]. To overcome a limitation of ineffective induction of NA immunity by seasonal vaccination, physical separation of HA and NA immunization was shown to induce IgG responses to HA and NA, avoiding competition of intravironic antigens [24]. The differential contribution of NA immunity to homologous and heterologous protection within the same NA subtype viruses was reported with adjuvanted recombinant NA protein (10 μg) vaccination in mice [9]. N1 NA (A/PR8, H1N1) could provide complete protection against homologous virus from morbidity and mortality, but lower efficacy of cross protection against heterologous H1N1 (2009 pandemic) and avian H5N1 viruses as evident by severe weight loss [9]. Reduced heterologous protection was similarly observed with recombinant N2 NA protein vaccination in mice, consistent with NA antigenic drifts, and heterosubtypic NA protection was not induced by monomeric NA vaccination [9], which is consistent in other studies reporting lower heterologous cross protection by NA-immunity after intramuscular vaccination [10]. Our prior studies demonstrated broader cross protection against heterosubtypic viruses by 5×M2e VLP vaccine, although M2e immunity alone has a limitation of low efficacy [18,25]. To further overcome NA antigenic drifts and we investigated new approaches to improve the breadth and efficacy of NA plus M2e immunity, consensus NA sequences were designed from the isolates after 2010 and implemented in the full-length NA constructs. The VLP platform expressed in insect cells has a unique feature to incorporate multiple NA proteins (cN1, cN2, and B cNA) in a membrane anchoring form, mimicking viral surface glycoproteins, and covering both seasonal influenza A and B viruses. Additionally, 5×M2e tandem repeat was incorporated into the same multi-NA VLP format using a multi-gene expressing baculovirus vector [26]. Monomeric N1 NA VLP vaccines were shown to induce protective immunity against homologous virus in ferrets [10] and protection against homologous and heterologous viruses in mice [11]. Influenza HA (2009 H1N1, H5N1, H7N9) VLP vaccines produced in insect cells were safe and efficacious in clinical trials [27–29], suggesting VLP as a feasible vaccine platform delivering multi-NA and M2e immunogens.

m-cNA-M2e VLP vaccination induced NAI activities, a known correlate of NA immunity, against a broad range of viruses including heterologous and heterosubtypic H1N1, H5N1, H3N2, H1N2, H7N9, and H9N2. Challenge viruses were heterologous since multi cNA-M2e VLP vaccine contains multi-NA proteins with artificial consensus sequences. m-cNA-M2e VLP vaccination protected mice against A/Nanchang/1995 and rgA/H1N2 (N2 of A/Switzerland/2013) viruses containing NA that has high homology (93% and 98%) with cN2 (S7A Fig). Even for N2 viruses with phylogenetically more distant 81% to 85% homology with cN2, m-cNA-M2e VLP vaccination could confer broad protection against cross-subtype N2-expressing viruses (H3N2 and H9N2) despite moderate weight loss. In contrast, monomeric cN2 VLP conferred protection against rgH1N2 virus (98% homology) but did not provide protection against drifted H3N2 (A/HK/1968 and A/Phil/1982) and H9N2 (rgA/HK/1999 H9N2) viruses, suggesting the contribution of 5×M2e in m-cNA-M2e VLP to inducing protection against NA drifted viruses. Significant protection against H1N1, rgA/VN H5N1, and rgA/SH H7N9 viruses was induced by vaccination with m-cNA-M2e VLP but not with monomeric cN2 VLP vaccination, consistent with the impact of m-cNA-M2e immunity on broadening the cross protection. In future studies, it is important to test the protective efficacy of this multi-NA-M2e construct against influenza A viruses containing heterologous avian M2e sequences. The monomeric cN1 VLP was similarly effective in inducing protection against rgA/VN H5N1 virus with 85% NA homology (S7B Fig). In particular, the contribution of 5×M2e appears to be significant as shown by 5×M2e VLP-induced protection against rgA/VN H5N1 virus at a comparable level.

In aged mice, m-cNA-M2e VLP was immunogenic in inducing humoral and cellular responses to M2e and NA, and provided 100% cross protection against H1N1, H3N2, and rgH7N9 viruses, preventing severe weight loss. Nonetheless, limitations are also noted in the aged mouse model in this study. The mouse age of 14 months old is estimated to be comparable to be approximate 50 years old of late middle-aged adults, which does not represent elderly populations. Most human individuals have pre-existing immunity to prior influenza vaccination or infection, which should be an important consideration for interpreting the data outcomes of immunogenicity and protective efficacy of vaccination in the aged mouse model of influenza.

The NA genetic diversity appears to be limited in influenza B viruses [30]. Consistently, adjuvanted recombinant NA vaccination of influenza B virus was also reported to provide cross-lineage protection [9]. Influenza B virus consensus NA (B cNA) shows high homology (96%) with both lineages of B viruses (S7C Fig). Prominent protection against weight loss and mortality was observed in mice vaccinated with m-cNA-M2e VLP or monomeric B cNA VLP after lethal dose challenge with Victoria or Yamagata lineage viruses, correlating with broadly cross-reactive NAI activities. Seasonal inactivated influenza vaccination is not effective in inducing NA immunity [23]. First time, this study has significance of supporting evidence that a single entity of m-cNA-M2e VLP could be developed as a universal vaccine protecting both lineage influenza B viruses and antigenically distinct influenza A viruses.

The mechanisms of NA and M2e immunity are not fully understood. NAI antibodies prevent the release of budding viral particles on the infected cells and, to a less degree, interfere with effective entry at the mucosal sites and HA-mediated membrane-fusion by inhibiting NA enzymatic activity [5,31,32]. Other effector functions of non-neutralizing antibodies include ADCC and antibody-dependent cellular phagocytosis, known to play a role in M2e [33] and partially contributing to NA antibody immunity [34–36]. Prior studies demonstrated the roles of Fc receptors (FcRs) and macrophages in M2e immunity [33]; roles of FcRs in NA immunity remain to be further investigated since FcRs was not required for NA immune-mediated protection [37]. M2e and NA are known to contain CD4^+^ and CD8^+^ T-cell epitopes in mice and in humans [38–40] and depletion of T cells particularly CD4 T cells severely lowered the efficacy in m-cNA-M2e VLP immunized mice. Vaccination with m-cNA-M2e VLP could induce systemic and mucosal IgG and IFN-γ secreting T cell responses to both M2e and NA, which play a role in broadening and enhancing cross protection. NA immunity in respiratory mucosal sites might be contributing to better protection in mice [9] and protecting against influenza B virus transmission in Guinea Pigs [41], suggesting that mucosal delivery of m-cNA-M2e VLP vaccine might provide more effective cross protection.

In summary, we presented data supporting that m-cNA-M2e VLP has the capacity to induce immunity to M2e and multi-subtype NA of influenza A and B viruses, and broad cross protection against morbidity and mortality under lethal challenges in mice. Developing m-cNA-M2e VLP as a new universal vaccine candidate, this study provides a supportive proof-of-concept approach to overcome a limitation of NA antigenic drifts in broadening cross protection and extend cross immunity to both influenza A N1 and N2 major subtypes, heterologous and heterosubtypic viruses, and influenza B NA, in addition to M2e. In an outbreak of HA variants or pandemic, immunity to broad NA and universal M2e epitopes is expected to provide protection against severe disease and mortality. Nonetheless, protective immunity by m-cNA-M2e VLP is not sterilizing, permitting a certain level of viral replications in the lung due to the non-neutralizing nature of NA and M2e immunity. Permissive protection was reported to provide immunologic benefits of effectively protecting future pandemics [42–44]. An alternative will be to supplement seasonal HA-based vaccines with m-cNA-M2e VLP as reported with purified recombinant NA [45] or 5×M2e VLP [39] or to test the efficacy of m-cNA-M2e VLP under pre-existing immunity, mimicking the general human population. Overall, this study warrants further testing of m-cNA-M2e VLP as a universal vaccine candidate such as in relevant ferret animals.

## Materials and methods

### Ethics statement

Mouse studies were approved by Georgia State University (GSU) Institutional Animal Care and Use Committee (IACUC, A21004) and carried out with the Guide for the Care and Use of Laboratory Animals of the National Institute of Health (NIH). Young and aged BALB/c mice were purchased from Jackson Laboratory and Taconic respectively, were housed in the animal facility at GSU.

### Influenza genes and recombinant baculovirus (rBV) constructs

Consensus NA (cN1 NA, cN2 NA, influenza B cNA) sequences (S1 Fig) were obtained from aligning the NA sequences of the human isolates (2010–2019) available at NCBI by using the sequence alignment program (UGENE software) (32). NA and 5×M2e genes were codon-optimized (S1 Fig) for high-level expression in Sf9 insect cells and synthesized (Gen-script, Piscataway, NJ). The 5×M2e construct consists of M2e from human, swine, and type I/II avian influenza A viruses as previously detailed (21). A plasmid DNA (pFastBac1) was engineered to express 3 full-length NA genes (cN1 NA, cN2 NA, influenza B cNA), 5×M2e, and M1 genes in tandem under each transcriptional polyhedrin promoter (Fig 1A) as previously described (33–35). The 5 genes to be expressed were introduced into the single pFastBac1 transfer vector and confirmed for correct insertions. A baculovirus expressing 5 genes (m-cNA-M2e, Fig 1A) was generated by using the Bac-to-Bac expression system, and plaques were purified and amplified, and high titer stocks were prepared and confirmed using gene specific PCR (Fig 1B). To prepare cNA or 5×M2e VLP, bacmid DNA containing each consensus NA or 5×M2e gene were isolated from DH10Bac *E*. *coli* after cloning into pFastBac and used to transfect Sf9 cells to generate mono-expressing baculovirus as described (13, 21).

### Expression and characterization of consensus multi-subtype m-cNA-M2e VLPs

Influenza VLP vaccines were produced as previously described (13). Sf9 cells maintained in suspension cultures of SF900II-SFM serum free medium were infected with baculovirus expressing consensus monomeric NA and 5×M2e, or multi 5 genes (cN1, cN2, B-cNA, 5×M2e, M1). VLPs were harvested from the culture supernatants containing released VLPs by low-speed centrifugation (2,000 ×g) to remove cell debris, then purified by ultracentrifugation (100,000 ×g) and resuspended in phosphate buffered saline (PBS). The protein concentration of VLPs were quantified by a protein assay kit (Bio-rad, Irvine, CA) and characterized by ELISA and western blot using M2e mAb 14C2, pan NA HCA-2 mAb, or M1 specific mAb (ab22396, Abcam). *S*odium dodecyl sulfate–polyacrylamide *gel* electrophoresis (SDS-PAGE) was performed using 4–12% gradient polyacrylamide gels (Invitrogen). Nanoparticle size distribution of VLPs was determined by dynamic light scattering (DLS) with a Malvern Zetasizer Nano ZS (Malvern Instruments, Westborough, MA). The functional activity for NA expressed on the surface of VLPs was determined by enzyme linked lectin assay (ELLA) as described (13, 36).

### Immunization and challenge of mice

Female young (6- to 8-week-old) and aged (14-month-old) BALB/c mice were IM immunized twice, at a 3-week interval, with 100 μl (50 μl in left and in right leg) of VLPs; 10 μg of m-cNA-M2e VLP, 3 μg of cN1 VLP, 3 μg or 10 μg of cN2 VLP, 3 μg of B cNA VLP, 3 μg of 5×M2e VLP. Use of a higher dose (10 μg) for m-cNA-M2e VLP than that (3 μg) of monomeric VLP was based on the observation that m-cNA-M2e VLP showed 10-fold lower reactivity than monomeric 5×M2e VLP (Fig 1G and 1H). PBS was used as a mock control. Bloods were collected at 2 weeks after prime and boost immunization. After boost, mice were challenged intranasally with a lethal dose of influenza A and B viruses in 50 μl PBS. Body weight changes and survival rates were monitored daily for 14 days.

The influenza viruses used for challenge were as follows; A/California/04/2009 H1N1 (A/Cal H1N1), mouse adapted A/Fort Monmouth/1/1947 H1N1 (A/FM H1N1), rgA/VN H5N1 containing H5 HA with the polybasic cleavage site deleted and N1 NA derived from A/Vietnam/1203/2004 and the backbone genes from A/Puerto Rico/8/1937 (A/PR8 H1N1) (37). The rgA/H1N2 virus contains N2 NA derived from A/Switzerland/2013 H3N2 and the remaining seven genes from A/PR8 (38), A/Philippine/2/1982 H3N2 (A/Phil), rgA/NC H3N2 with H3 HA and N2 NA from A/Nanchang/933/1995 and A/PR8 backbone, A/Hong Kong/1/1968 H3N2 (A/HK H3N2), A/Hong Kong/1073/99 H9N2 (A/HK H9N2), rgA/SH H7N9 containing H7 HA and N9 NA from A/Shanghai/02/2013 and A/PR8 backbone (39). For influenza B virus challenges, we used Victoria lineage B/Malaysia/2056/2004 (B/ML) and Yamagata lineage B/Florida/4/2006 (B/FL). All viruses were propagated in embryonated chicken eggs, and 50% egg-infectious doses (EID_50_) were determined using 0.5% chicken red blood cells.

### Enzyme-linked immunosorbent assay (ELISA)

ELISA virus antigens include N1 NA (A/Cal, H1N1, BEI, NR-19234), N2 NA (A/Brisbane/10/2007 H3N2, BEI, NR-43784), influenza B NA proteins (B/Florida/4/2006, BEI, NR-19236), human M2e (hM2e, SLLTEVETPIRNEWGSRSN) peptide, and inactivated influenza A viruses. Serially diluted immune sera were applied onto 96-well microtiter plates pre-coated with antigens at 200 ng/mL of N1, N2 NA, influenza B/FL NA proteins, 4 μg/mL of hM2e peptide and inactivated influenza viruses. For assays of IgG antibody-secreting mediastinal lymph node (mLN) and spleen cell responses, the cells isolated were *in vitro* cultured for 1 (D1) or 5 days (D5) on the plate precoated with influenza virus antigens. The combined levels of IgG antibodies secreted into the culture supernatants and captured on the plate were analyzed. The IgG and IgG isotypes were determined using horseradish peroxidase (HRP)-conjugated goat anti-mouse IgG, IgG1, IgG2a secondary antibodies (SouthernBiotech, Birmingham, AL) and tetramethylbenzidine (TMB) (eBiosciences, San Diego, CA), and antibody levels are presented as optical density (OD) at 450 nm (BioTeck ELISA plate reader) or concentrations as calculated using standard IgG (Southern Biotech) as previously described (13). The ELISA OD values of IgG were converted to quantitative concentrations of IgG antibodies, based on the standard curve equations of purified IgG antibodies commercially available (Southern Biotech, Birmingham, AL).

### Enzyme-linked lectin assay (ELLA)

NA inhibition (NAI) activity of immune sera against influenza virus was determined by ELLA using a fetuin-based procedure as described (13, 36, 40). Briefly, virus and immune sera were added to 96-well plates coated with 25 μg/mL of fetuin (Sigma-Aldrich) and then the plates were incubated at 37°C for 20 h. After incubation with 1 μg/mL of HRP-labeled peanut lectin, NAI activity was measured by using TMB substrate (eBiosciences) to develop colorimetric reaction. The inhibition percentage was calculated using the formula: 100 × (OD_virus only control_—OD_test sample_)/OD_virus only control_.

### Lung viral titration

Lung viral titers were determined in embryonated chicken eggs. Ten-fold serial dilutions of lung lysates were injected into 10-day embryonated chicken eggs and then incubated for 3 days. Virus titers were determined by hemagglutination assay of the allantoic fluids. The titers of EID_50_ were determined according to the Reed and Muench method (41).

### ELISpot assay

IFN-γ secreting cells were evaluated in the lung and spleen samples by enzyme-linked immunospot (ELISpot) analysis as described previously (42). Stimulating antigens used for ELISpot assay were M2e peptide and NA peptide pools (A/Brisbane/10/2007 H3N2). Lung (5×10^5^ cells/well) and spleen cells (10^6^ cells/well) were cultured on 96-well ELISpot plates precoated with anti-mouse IFN-γ capture antibody (BD Biosciences, San Diego, CA) in the presence of 5 μg/mL peptides or virus antigens. The spots were developed with biotinylated anti-mouse IFN-γ antibody and alkaline phosphatase-labeled streptavidin (BD Pharmingen), visualized with a 3,3’-diaminobenzidine substrate, and counted by an ELISPOT reader (BioSys, Miami, FL).

### Flow cytometry

Single cell suspensions for flow cytometry analysis were prepared on 44/67% Percoll gradient after homogenizing the lung tissues collected day 6 or 5 post challenge as described (42). Then, the cells were *in vitro* stimulated with 5 μg/mL of M2e peptide or NA [A/Brisbane/10/2007 H3N2 (BEI, NR-19251) and A/Vietnam/1203/2004 H5N1 (BEI, NR-19258) peptide pools in presence of Brefeldin A (20 μg/mL) for 5 h at 37°C. The lymphocytes were stained with anti-mouse CD3 (clone 17A2, BD, San Diego, CA), CD4 (clone 553051, BD), CD8 (clone 25-0081-82, eBiosciences, San Diego, CA), and IFN-γ (clone XMG1.2, BD) mAb. Intracellular cytokine staining of lymphocytes was followed by using BD Cytofix/Cytoperm Plus kit. Cytokine-expressing cells were acquired on a Becton-Dickinson LSR-II/Fortessa flow cytometer and analyzed by Flowjo software (Tree Star, Inc., Ashland, OR).

### *In vivo* protection test of immune sera

For immune passive transfer experiments, sera (25 μl) after heat-inactivation were mixed with 25 μl of 4×LD_50_ A/Phil and incubated at room temperature for 30 min as described (21). A mixture of A/Phil H3N2 virus and sera was intranasally administered to naïve BALB/c mice, and body weight changes and survival rates were monitored daily for 14 days.

### Antibody-dependent cellular cytotoxicity (ADCC) assay

ADCC activity of immune sera was performed according to the manufacturer’s protocol (Promega). Briefly, Madin-Darby Canine Kidney cells (MDCKs, ATCC) maintained in Dulbecco’s Modified Eagle Medium media (DMEM) supplemented with 10% heat inactivated fetal bovine serum were seeded in sterile white 96 well plates. The MDCKs on the 96-well plates were infected with 100×TCID_50_ of influenza A viruses a day prior to assay. Immune sera diluted in assay buffer and effector Jurkat cells (43) expressing mouse FcγRIV (Promega) were added to virus-infected MDCK target cells and then incubated for 6 h. Luminescence was read on a Cytation 5 imaging reader (BioTek) after 5 min incubation with 75 μL of Bio-Glo luciferase assay substrate (Promega).

### *In vivo* depletion of T cells

To deplete CD4 or CD8 T cells, BALB/c mice immunized with m-cNA-M2e VLP were injected intraperitoneally (i.p.) with 200 μg of anti-CD4 mAb (clone GK1.5, BioXCell) or 150 μg of anti-CD8 mAb (clone 53.6.7, BioXCell) on day -2 and +2 before/after challenge as previously described (44). Over 99% depletion efficacy of CD4 and CD8 T cells was confirmed by flow cytometry of blood samples (Fig 8D).

### Statistical analysis

Data are represented as mean ± standard errors of the mean (SEM). The statistical significance was performed by one-way ANOVA with Tukey’s multiple comparison post-test and by two-way ANOVA with Bonferroni posttests. P values of less than 0.05 (*p*<0.05) were considered statically significant. Data were analyzed using a Prism software (GraphPad Software, Inc., San Diego, CA).

## Supporting information

S1 Fig Amino acid sequence for consensus NA and tandem repeat 5×M2e. (PDF)

S2 Fig Neuraminidase (NA) inhibition activity in percentages by immune sera. NA inhibition activities were measured from serially diluted boost immune and naïve sera by ELLA.
(PDF)

S3 Fig Comparison of humoral immune responses in boost antisera between m-cNA-M2e VLP and monomeric VLP mix in mice. BALB/c mice (n = 10 per group) were immunized twice with m-cNA-M2e VLP (10 μg) or VLP mix (12 μg of VLP mix: cN1 VLP 3μg +cN2 VLP 3μg +5xM2e VLP 3μg +B-NA VLP 3μg). Humoral immune responses were determined in boost antisera. Concentrations (μg/mL) of IgG antibodies specific for M2e (A) and N2 NA (B) as determined by ELISA. (C-E) Concentrations (μg/mL) of IgG antibodies specific for inactivated viruses as indicated: (C) IgG to inactivated A/California/2009 H1N1, (D) IgG to inactivated A/Switzerland/2013 H3N2, (E) IgG to inactivated A/Wisconsin/2009 H3N2. (F-G) NA inhibition (NAI) in percentages at 40X and 200X serum dilutions. (F) NAI to A/California/2009. (G) NAI to A/Switzerland/2013. Data represented as mean ± SEM; statistical significances were performed by one-way ANOVA with Tukey’s multiple comparison test and indicated as **, *P* < 0.01; ***, *P* < 0.001; ns, no significant difference between compared groups.
(PDF)

S4 Fig IFN-γ-secreting cellular and IgG antibody secreting and humoral immune responses in mice with m-cNA-M2e VLP vaccination. Immunized mice (n = 4 per group, 6–8 weeks old adult mice) were infected with A/Phil/1982 (H3N2) virus. **(A and B)** IFN-γ-secreting cell spots. Splenocytes and lung cells were cultured on the ELISpot plate pre-coated with cytokine capture antibody in the presence of 5 μg/mL of (A) M2e or (B) N2 NA (A/Brisbane/10/2007 H3N2) peptide pools. **(C and D)** Antigen-specific IgG antibodies were determined from mediastinal lymph node (mLN) and spleen harvested on day 6 post infection and subsequent *in vitro* culture for 5 days (D5) on the plate precoated with 2 μg/mL of (C) M2e peptide or 200 ng/mL of (D) N2 NA protein (A/Brisbane/10/2007 H3N2). The statistical significances were performed with one-way ANOVA with Tukey’s Multiple Comparison test and indicated as *, *P* < 0.05; **, *P* < 0.01; ***, *P* < 0.001 between indicated groups.
(PDF)

S5 Fig IgG antibody levels in respiratory mucosal sites and reduced lung inflammatory cytokines in young adult mice with m-cNA-M2e vaccination upon influenza A virus infection. Young adult mice (n = 4 per group, 6–8 weeks old adult mice) were vaccinated with m-cNA-M2e VLP, cN2 VLP, or 5xM2e VLP. (**A and B)** IgG levels (ng/mL) specific for (A) M2e peptide or (B) NA2 protein (A/Brisbane/10/2007 H3N2) in the bronchoalveolar lavage fluid (BALF) and lung lysates harvested on day 6 post infection with A/Phil H3N2 virus. (**C and D)** The levels of IFN-γ and IL-6 in BALF and lung extracts by ELISA. Data represented as mean ± SEM; statistical significances were performed by one-way ANOVA with Tukey’s multiple comparison test and indicated as *, *P* < 0.05; **, *P* < 0.01; ***, *P* < 0.001; ns, no significant difference between compared groups.
(PDF)

S6 Fig IgG antibody levels in respiratory mucosal sites and reduced lung inflammatory cytokines in aged mice with m-cNA-M2e vaccination upon influenza A virus infection. Aged BALB/c mice (14-month-old, n = 4 per group) were vaccinated with m-cNA-M2e VLP. (**A and B)** IgG levels (ng/mL) specific for (A) M2e peptide or (B) NA2 protein (A/Brisbane/10/2007 H3N2) in BALF and lung lysates harvested on day 6 post infection with A/Phil H3N2 virus. (**C and D)** The levels of IFN-γ and IL-6 in BALF and lung extracts by ELISA. Data represented as mean ± SEM; statistical significances were performed by one-way ANOVA with Tukey’s multiple comparison test and indicated as *, *P* < 0.05; **, *P* < 0.01; ***, *P* < 0.001; ns, no significant difference between compared groups.
(PDF)

S7 Fig Sequence homology between the consensus NA vaccines and influenza viruses used for challenge. The sequence similarity was identified using basic local alignment search tool (BLAST) with protein BLAST. (**A)** Sequence homology between the consensus N1 (cN1) and N2 (cN2) NA vaccines and influenza viruses containing N2 NA. **(B)** Sequence homology between the consensus N1 (cN1) and N2 (cN2) NA vaccines and influenza viruses containing N1 or N9 NA. **(C)** Sequence homology between the consensus influenza B NA (B cNA) vaccine and influenza B viruses. NA GenBank ID: AFG72628 for A/Nanchang (H3N2), ABQ97206 for A/Hong Kong/1/1968 (H3N2), AAO46474 for A/Philippine/1982 (H3N2), NP_859038 for A/Hong Kong (H9N2), ADN89559 for A/California/2009 (H1N1), AAF77037 for A/Fort Monmouth/1947 (H1N1), AAT73329 for A/Vietnam/1203/2004 (H5N1), YP_009118481 for A/Shanghai/02/2013 (H7N9), ACA33351 for B/Florida/4/2006, AAO38878 for B/Hong Kong/330/2001, ACO05961 for B/Malaysia/2056/2004.
(PDF)
