# Supplementary material for: Universal protection against influenza viruses by multi-subtype neuraminidase and M2 ectodomain virus-like particle
Source: PLoS Pathog. 2022 Aug 25;18(8):e1010755. doi: 10.1371/journal.ppat.1010755 (PMC9409530; doi:10.1371/journal.ppat.1010755)
Supplement: S1 Fig — (PDF) [file ppat.1010755.s001.pdf]

Consensus N1 NA (cN1)

MNPNQKIITIGSVCMTIGMANLILQIGNIISIWVSHSIQIGNQSQIETCNQSVITYENNT
WVNQTYVNISNTNFAAGQSVVSVKLAGNSSLCPVSGWAIYSKDNSVRIGSKGDVFVIREP
FISCSPLECRTFFLTQGALLNDKHSNGTIKDRSPHRTLMSCPIGEAPSPYNSRFESVAWS
ASACHDGTSWLTIGISGPDSGAVAVLKYNGIITDTIKSWRNNILRTQESECACVNGSCFT
IMTDGPSDGQASYKIFKMEKGKIVKSVEMDAPNYHYEECSCYPDSSEITCVCRDNWHGSN
RPWVSFNQNLEYQIGYICSGVFGDNPRPNDKTGSCGPVSSNGANGVKGFSFKYGNGVWIG
RTKSTSSRKGFEMIWDPNGWTGTDNKFSIKQDIVGINEWSGYSGSFVQHPELTGLDCIRP
CFWVELIRGRPEENTIWTSGSSISFCGVNSDTVGWSWPDGAELPFTIDK

Consensus N2 NA (cN2)

MNPNQKIITIGSVSLTISTICFFMQIAILITTVTLHFKQYEFNSPPNNQVMLCEPTIIER
NITEIVYLTNTTIEKEICPKPAEYRNWSKPQCGITGFAPFSKDNSIRLSAGGDIWVTREP
YVSCDPDKCYQFALGQGTTLNNVHSNDTVRDRTPYRTLLMNELGVPFHLGTKQVCIAWSS
SSCHDGKAWLHVCITGDDKNATASFIYNGRLVDSVVSWSKDILRTQESECVCINGTCTVV
MTDGSASGKADTKILFIEEGKIVHTSKLSGSAQHVEECSCYPRYPGVRCVCRDNWKGSNR
PIVDINIKDHSIVSSYVCSGLVGDTPRKNDSSSSSHCLDPNNEEGGHGVKGWAFDDGNDV
WMGRTISEKSRSGYETFKVVEGWSNPKSKLQINRQVIVDRGDRSGYSGIFSVEGKSCINR
CFYVELIRGRKEETEVLWTSNSIVVFCGTSGTYGTGSWPDGADLNLMPI

Consensus influenza B NA (B cNA)

MLPSTIQTLTLFLTSGGVLLSLYVSASLSYLLYSDILLKFSRTEITAPIMPLDCANASNV
QAVNRSATKGVTPLLPEPEWTYPRLSCPGSTFQKALLISPHRFGETKGNSAPLIIREPFI
ACGPKECKHFALTHYAAQPGGYYNGTREDRNKLRHLISVKLGKIPTVENSIFHMAAWSGS
ACHDGKEWTYIGVDGPDSNALLKIKYGEAYTDTYHSYAKNILRTQESACNCIGGDCYLMI
TDGPASGVSECRFLKIREGRIIKEIFPTGRVKHTEECTCGFASNKTIECACRDNSYTAKR
PFVKLNVETDTAEIRLMCTKTYLDTPRPNDGSITGPCESDGDKGSGGIKGGFVHQRMASK
IGRWYSRTMSKTKRMGMGLYVKYDGDPWTDSEALALSGVMVSMEEPGWYSFGFEIKDKKC
DVPCIGIEMVHDGGKTTWHSAATAIYCLMGSGQLLWDTVTGVNMTL

5×M2e (Human (x2)-Swine-Avian I/II)

MKFLVNVALVFMVVYISYIYADPINMTTSINNNLQRVRELAVQSANSAAAPGAAVDGTSL
LTEVETPIRNEWGSRSNDSSDAAAGGAASLLTEVETPIRNEWGSRSNDSSDAAAPGAASL
LTEVETPTRSEWESRSSDSSDAAAGGAASLLTEVETPTRNEWESRSSDSSDAAAPGAASL
LTEVETLTRNGWGCRCSDSSDGGLKQIEDKLEEILSKLYHIENELARIKKLLGELEILAI
YSTVASSLVLLVSLGAISFWMCSNGSLQCRICI

S1 Figure. Amino acid sequence for consensus NA and tandem repeat 5×M2e.
