# Supplementary material for: Universal protection against influenza viruses by multi-subtype neuraminidase and M2 ectodomain virus-like particle
Source: PLoS Pathog. 2022 Aug 25;18(8):e1010755. doi: 10.1371/journal.ppat.1010755 (PMC9409530; doi:10.1371/journal.ppat.1010755)
Supplement: S2 Fig — NA inhibition activities were measured from serially diluted boost immune and naïve sera by ELLA. (PDF) [file ppat.1010755.s002.pdf]

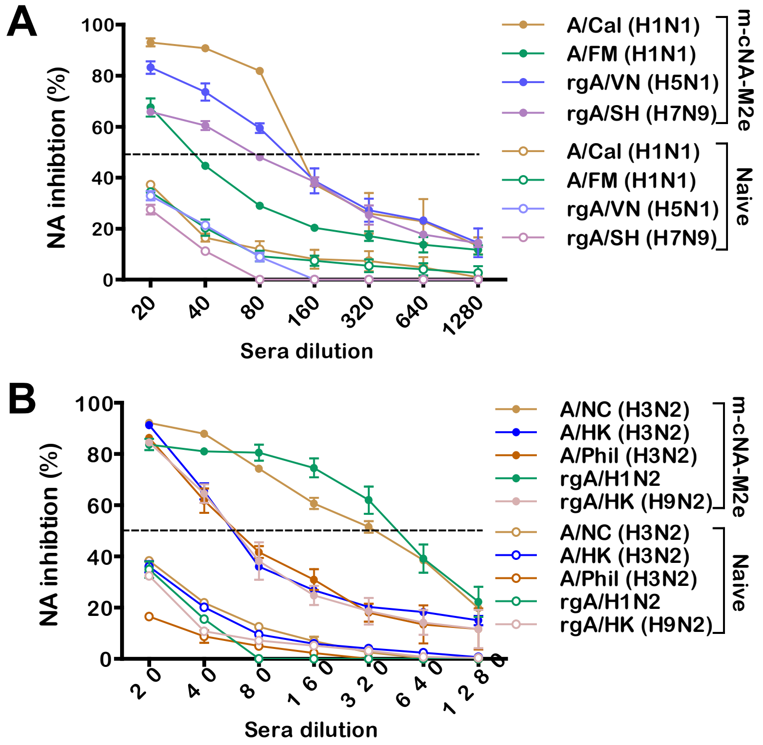

S2 Figure. Neuraminidase (NA) inhibition activity in percentages by immune sera. NA inhibition activities were measured from serially diluted boost immune and naïve sera by ELLA.
