# Supplementary material for: Universal protection against influenza viruses by multi-subtype neuraminidase and M2 ectodomain virus-like particle
Source: PLoS Pathog. 2022 Aug 25;18(8):e1010755. doi: 10.1371/journal.ppat.1010755 (PMC9409530; doi:10.1371/journal.ppat.1010755)
Supplement: S3 Fig — BALB/c mice (n = 10 per group) were immunized twice with m-cNA-M2e VLP (10 μg) or VLP mix (12 μg of VLP mix: cN1 VLP 3μg +cN2 VLP 3μg +5xM2e VLP 3μg +B-NA VLP 3μg). Humoral immune responses were determined in boost antisera. Concentrations (μg/mL) of IgG antibodies specific for M2e (A) and N2 NA (B) as determined by ELISA. (C-E) Concentrations (μg/mL) of IgG antibodies specific for inactivated viruses as indicated: (C) IgG to inactivated A/California/2009 H1N1, (D) IgG to inactivated A/Switzerland/2013 H3N2, (E) IgG to inactivated A/Wisconsin/2009 H3N2. (F-G) NA inhibition (NAI) in percentages at 40X and 200X serum dilutions. (F) NAI to A/California/2009. (G) NAI to A/Switzerland/2013. Data represented as mean ± SEM; statistical significances were performed by one-way ANOVA with Tukey’s multiple comparison test and indicated as **, P < 0.01; ***, P < 0.001; ns, no significant difference between compared groups. (PDF) [file ppat.1010755.s003.pdf]

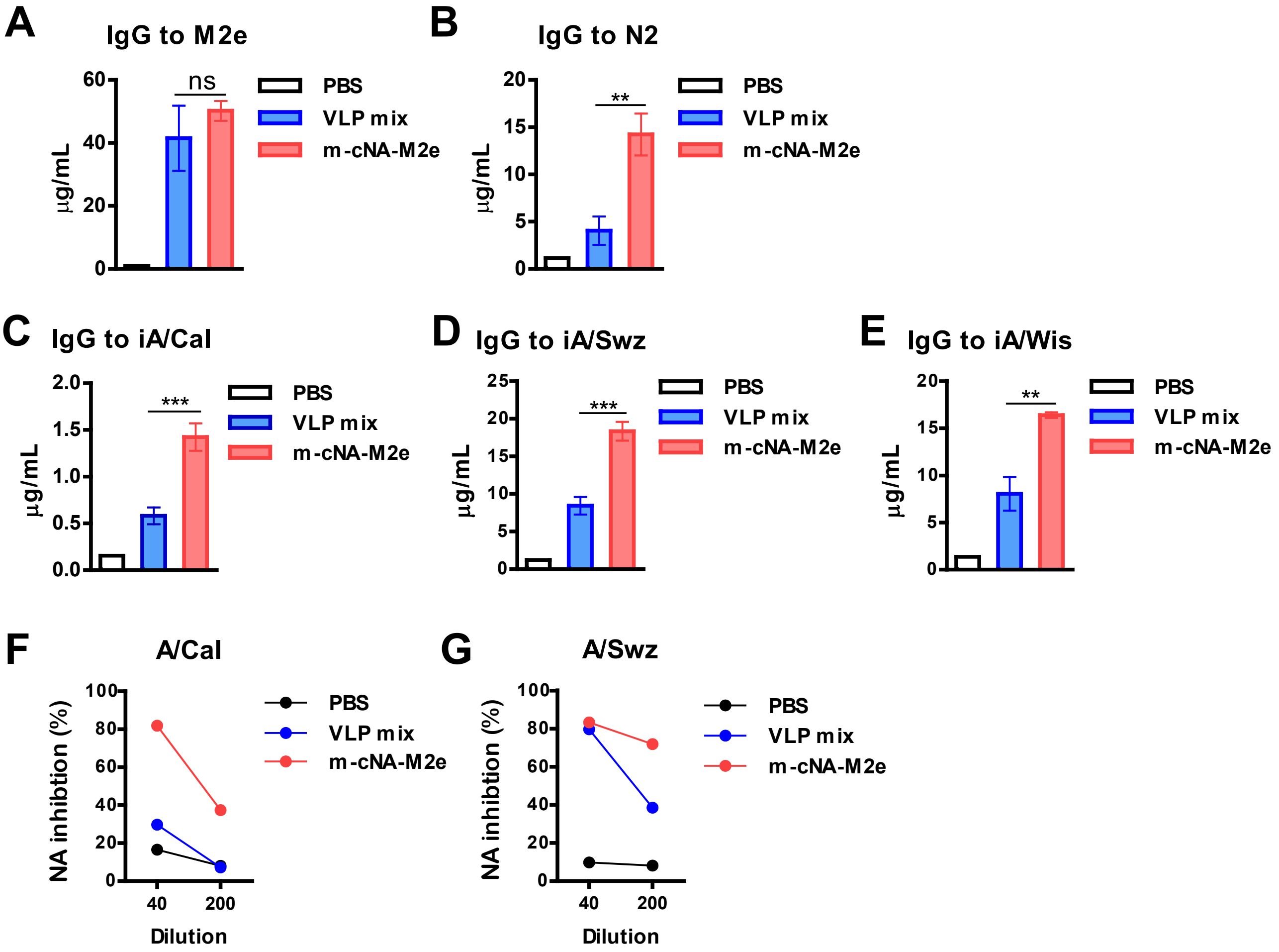

S3 Figure. Comparison of humoral immune responses in boost antisera between m-cNA-M2e VLP and monomeric VLP mix in mice. BALB/c mice (n=10 per group) were immunized twice with m-cNA-M2e VLP (10 µg) or VLP mix (12 µg of VLP mix: cN1 VLP 3µg +cN2 VLP 3µg +5xM2e VLP 3µg +B-NA VLP 3µg). Humoral immune responses were determined in boost antisera. Concentrations (µg/mL) of IgG antibodies specific for M2e (A) and N2 NA (B) as determined by ELISA. (C-E) Concentrations (µg/mL) of IgG antibodies specific for inactivated viruses as indicated: (C) IgG to inactivated A/California/2009 H1N1, (D) IgG to inactivated A/Switzerland/2013 H3N2, (E) IgG to inactivated A/Wisconsin/2009 H3N2. (F-G) NA inhibition (NAI) in percentages at 40X and 200X serum dilutions. (F) NAI to A/California/2009. (G) NAI to A/Switzerland/2013. Data represented as mean ± SEM; statistical significances were performed by one-way ANOVA with Tukey's multiple comparison test and indicated as **, $P < 0.01$; ***, $P < 0.001$; ns, no significant difference between compared groups.
