# Supplementary material for: Universal protection against influenza viruses by multi-subtype neuraminidase and M2 ectodomain virus-like particle
Source: PLoS Pathog. 2022 Aug 25;18(8):e1010755. doi: 10.1371/journal.ppat.1010755 (PMC9409530; doi:10.1371/journal.ppat.1010755)
Supplement: S4 Fig — Immunized mice (n = 4 per group, 6–8 weeks old adult mice) were infected with A/Phil/1982 (H3N2) virus. (A and B) IFN-γ-secreting cell spots. Splenocytes and lung cells were cultured on the ELISpot plate pre-coated with cytokine capture antibody in the presence of 5 μg/mL of (A) M2e or (B) N2 NA (A/Brisbane/10/2007 H3N2) peptide pools. (C and D) Antigen-specific IgG antibodies were determined from mediastinal lymph node (mLN) and spleen harvested on day 6 post infection and subsequent in vitro culture for 5 days (D5) on the plate precoated with 2 μg/mL of (C) M2e peptide or 200 ng/mL of (D) N2 NA protein (A/Brisbane/10/2007 H3N2). The statistical significances were performed with one-way ANOVA with Tukey’s Multiple Comparison test and indicated as *, P < 0.05; **, P < 0.01; ***, P < 0.001 between indicated groups. (PDF) [file ppat.1010755.s004.pdf]

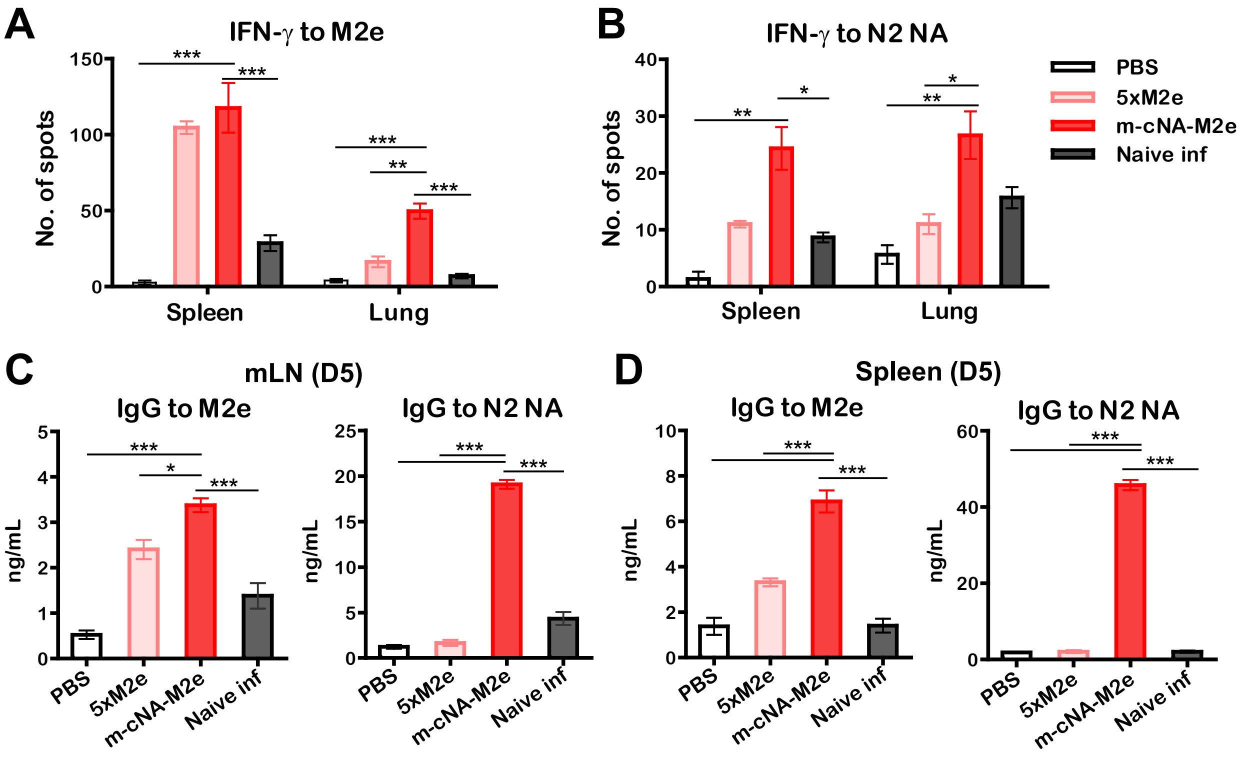

S4 Figure. IFN-γ-secreting cellular and IgG antibody secreting and humoral immune responses in mice with m-cNA-M2e VLP vaccination. Immunized mice (n=4 per group, 6-8 weeks old adult mice) were infected with A/Phil/1982 (H3N2) virus. **(A and B)** IFN-γ-secreting cell spots. Splenocytes and lung cells were cultured on the ELISpot plate pre-coated with cytokine capture antibody in the presence of 5 μg/mL of (A) M2e or (B) N2 NA (A/Brisbane/10/2007 H3N2) peptide pools. **(C and D)** Antigen-specific IgG antibodies were determined from mediastinal lymph node (mLN) and spleen harvested on day 6 post infection and subsequent *in vitro* culture for 5 days (D5) on the plate precoated with 2 μg/mL of (C) M2e peptide or 200 ng/mL of (D) N2 NA protein (A/Brisbane/10/2007 H3N2). The statistical significances were performed with one-way ANOVA with Tukey's Multiple Comparison test and indicated as *, $P < 0.05$; **, $P < 0.01$; ***, $P < 0.001$ between indicated groups.
