# Supplementary material for: Universal protection against influenza viruses by multi-subtype neuraminidase and M2 ectodomain virus-like particle
Source: PLoS Pathog. 2022 Aug 25;18(8):e1010755. doi: 10.1371/journal.ppat.1010755 (PMC9409530; doi:10.1371/journal.ppat.1010755)
Supplement: S5 Fig — Young adult mice (n = 4 per group, 6–8 weeks old adult mice) were vaccinated with m-cNA-M2e VLP, cN2 VLP, or 5xM2e VLP. (A and B) IgG levels (ng/mL) specific for (A) M2e peptide or (B) NA2 protein (A/Brisbane/10/2007 H3N2) in the bronchoalveolar lavage fluid (BALF) and lung lysates harvested on day 6 post infection with A/Phil H3N2 virus. (C and D) The levels of IFN-γ and IL-6 in BALF and lung extracts by ELISA. Data represented as mean ± SEM; statistical significances were performed by one-way ANOVA with Tukey’s multiple comparison test and indicated as *, P < 0.05; **, P < 0.01; ***, P < 0.001; ns, no significant difference between compared groups. (PDF) [file ppat.1010755.s005.pdf]

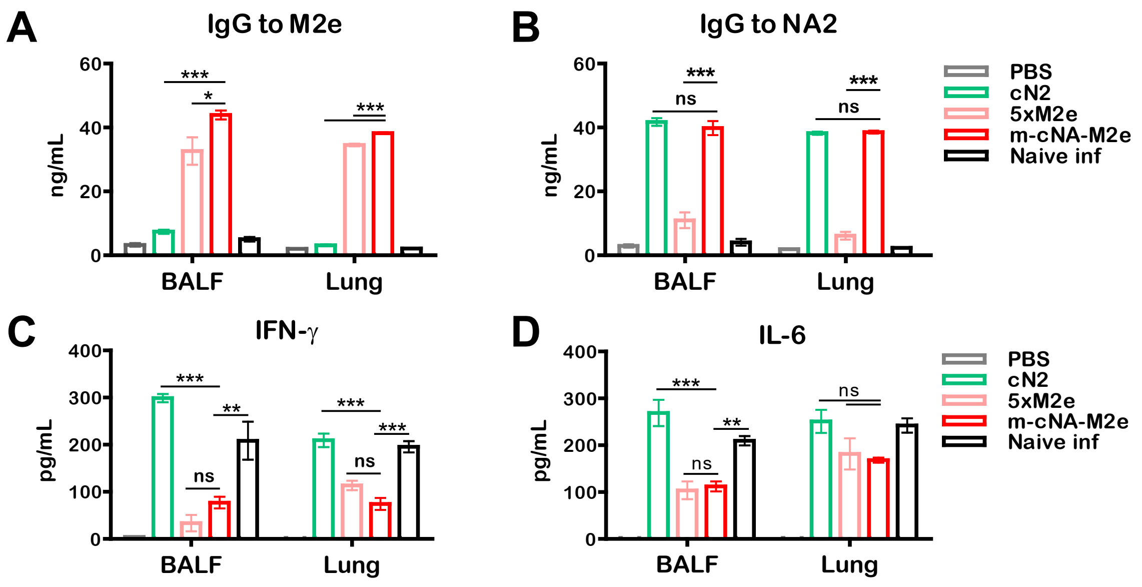

S5 Figure. IgG antibody levels in respiratory mucosal sites and reduced lung inflammatory cytokines in young adult mice with m-cNA-M2e vaccination upon influenza A virus infection. Young adult mice (n=4 per group, 6-8 weeks old adult mice) were vaccinated with m-cNA-M2e VLP, cN2 VLP, or 5xM2e VLP. (**A and B)** IgG levels (ng/mL) specific for (A) M2e peptide or (B) NA2 protein (A/Brisbane/10/2007 H3N2) in the bronchoalveolar lavage fluid (BALF) and lung lysates harvested on day 6 post infection with A/Phil H3N2 virus. (**C and D)** The levels of IFN-γ and IL-6 in BALF and lung extracts by ELISA. Data represented as mean ± SEM; statistical significances were performed by one-way ANOVA with Tukey's multiple comparison test and indicated as *, $P < 0.05$; **, $P < 0.01$; ***, $P < 0.001$; ns, no significant difference between compared groups.
