# Supplementary material for: Universal protection against influenza viruses by multi-subtype neuraminidase and M2 ectodomain virus-like particle
Source: PLoS Pathog. 2022 Aug 25;18(8):e1010755. doi: 10.1371/journal.ppat.1010755 (PMC9409530; doi:10.1371/journal.ppat.1010755)
Supplement: S7 Fig — The sequence similarity was identified using basic local alignment search tool (BLAST) with protein BLAST. (A) Sequence homology between the consensus N1 (cN1) and N2 (cN2) NA vaccines and influenza viruses containing N2 NA. (B) Sequence homology between the consensus N1 (cN1) and N2 (cN2) NA vaccines and influenza viruses containing N1 or N9 NA. (C) Sequence homology between the consensus influenza B NA (B cNA) vaccine and influenza B viruses. NA GenBank ID: AFG72628 for A/Nanchang (H3N2), ABQ97206 for A/Hong Kong/1/1968 (H3N2), AAO46474 for A/Philippine/1982 (H3N2), NP_859038 for A/Hong Kong (H9N2), ADN89559 for A/California/2009 (H1N1), AAF77037 for A/Fort Monmouth/1947 (H1N1), AAT73329 for A/Vietnam/1203/2004 (H5N1), YP_009118481 for A/Shanghai/02/2013 (H7N9), ACA33351 for B/Florida/4/2006, AAO38878 for B/Hong Kong/330/2001, ACO05961 for B/Malaysia/2056/2004. (PDF) [file ppat.1010755.s007.pdf]

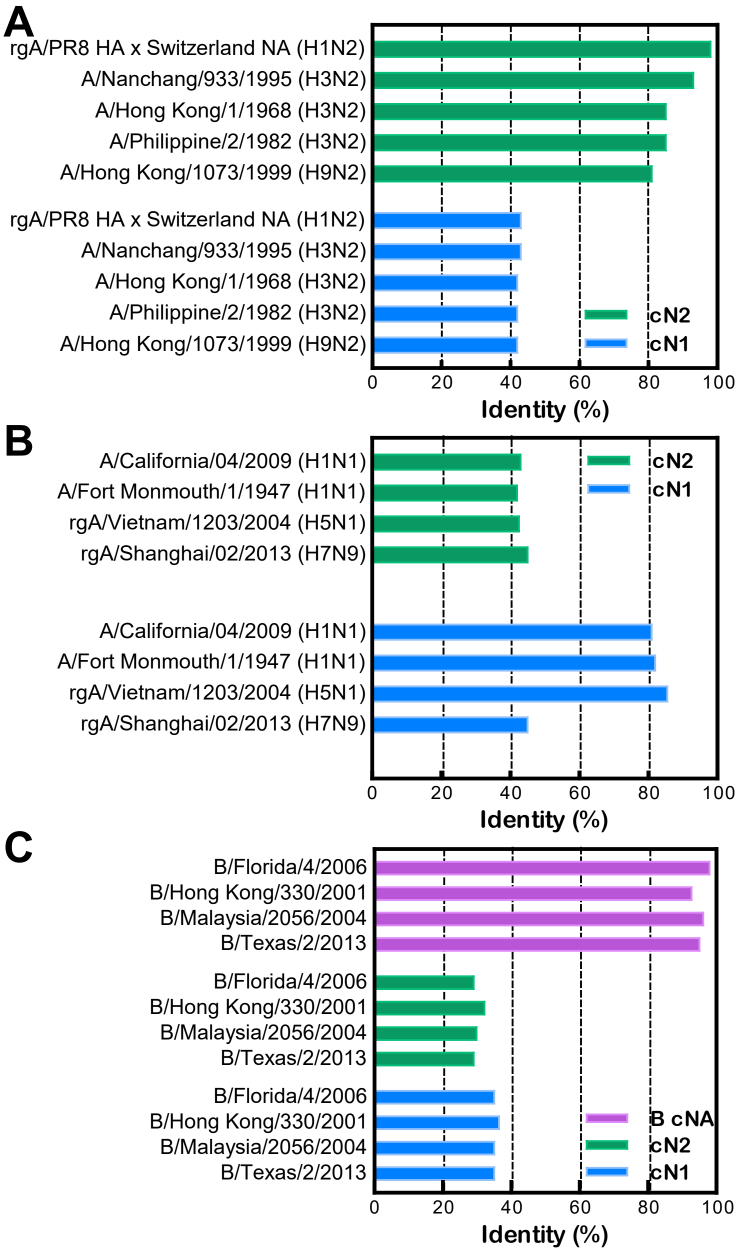

S7 Figure. Sequence homology between the consensus NA vaccines and influenza viruses used for challenge. The sequence similarity was identified using basic local alignment search tool (BLAST) with protein BLAST. (**A**) Sequence homology between the consensus N1 (cN1) and N2 (cN2) NA vaccines and influenza viruses containing N2 NA. **(B)** Sequence homology between the consensus N1 (cN1) and N2 (cN2) NA vaccines and influenza viruses containing N1 or N9 NA. **(C)** Sequence homology between the consensus influenza B NA (B cNA) vaccine and influenza B viruses. NA GenBank ID: AFG72628 for A/Nanchang (H3N2), ABQ97206 for A/Hong Kong/1/1968 (H3N2), AAO46474 for A/Philippine/1982 (H3N2), NP_859038 for A/Hong Kong (H9N2), ADN89559 for A/California/2009 (H1N1), AAF77037 for A/Fort Monmouth/1947 (H1N1), AAT73329 for A/Vietnam/1203/2004 (H5N1), YP_009118481 for A/Shanghai/02/2013 (H7N9), ACA33351 for B/Florida/4/2006, AAO38878 for B/Hong Kong/330/2001, ACO05961 for B/Malaysia/2056/2004.
